# Influence of face masks on the subjective impairment at different physical workloads

**DOI:** 10.1038/s41598-023-34319-0

**Published:** 2023-05-19

**Authors:** Vera van Kampen, Eike-Maximillian Marek, Kirsten Sucker, Birger Jettkant, Benjamin Kendzia, Bianca Strauß, Melanie Ulbrich, Anja Deckert, Hans Berresheim, Christian Eisenhawer, Frank Hoffmeyer, Simon Weidhaas, Thomas Behrens, Thomas Brüning, Jürgen Bünger

**Affiliations:** Institute for Prevention and Occupational Medicine of the German Social Accident Insurance, Institute of the Ruhr-University Bochum (IPA), Bürkle-de-la-Camp-Platz 1, 44789 Bochum, Germany

**Keywords:** Randomized controlled trials, Occupational health, Respiratory signs and symptoms, Health policy, Public health

## Abstract

To quantify the subjective and cognitive impairment caused by wearing face masks at work, 20 men and 20 women (median age 47 years, range 19–65) were tested under different ergometer workloads while wearing surgical mask, community mask, FFP2 respirator or no mask in a randomized and partially double-blinded design. Masks were worn also at the workplace for four hours. Subjective impairment was recorded by questionnaires. Cognitive performance was tested before and after the workplace examination. Subjective feeling of heat, humidity, and difficult breathing increased with rising physical exertion and wearing time for all three mask types, most notably for FFP2. Even when blinded, participants with FFP2 reported difficult breathing already at rest. During physical exertion, individuals with low tolerance to discomfort reported significantly stronger impairment (OR 1.14, 95% CI 1.02–1.27). Regarding light work, older subjects (OR 0.95, 95% CI 0.92–0.98) and women (OR 0.84, 95% CI 0.72–0.99) showed significantly lower and atopic subjects stronger impairment (OR 1.16, 95% CI 1.06–1.27). No significant influence of mask wearing was detected on cognitive performance. Wearing a mask had no effect on cognitive performance, but led to discomfort which increased with physical exertion and wearing time. Individuals who tolerate discomfort poorly felt more impaired by wearing a mask during physical exertion.

## Introduction

During the SARS-CoV-2 pandemic, in most countries face masks were recommended or mandatory in medical institutions, public areas, and at workplaces^1^. Depending on the mask type, mask wearing protects from virus transmission^2–4^. Filtering facepieces (respirators, e.g. N95, FFP2) provide a better protection efficiency than surgical masks (SM) and community masks (cloth masks, CM) due to their higher filtration efficiency and their ability to provide a better fit^5, 6^.

Wearing masks under physical exercise up to the maximum workload lead to cardiopulmonary stress of the tested subjects in several studies. Reviews^7–9^ summarized, that wearing a mask during physical activity may increase dyspnea but has little effect on work of breathing, blood gases and other physiological parameters, even during maximum exercise.

Shaw et al.^9^ also considered the subjects' perceived exertion in their review. In most studies, the Borg scale was used for rating of perceived exertion during physical activity and both, SM and N95 increased the perceived exertion significantly compared to the no mask situation. In contrast to this, other studies did not find significant differences in subjectively perceived exertion between mask wearing and no mask conditions^10, 11^. In another study, a distinction was made between perceived physical exertion and perceived respiratory exertion. While during ergometry there was no effect on the former, perceived respiratory exertion was significantly higher with SM, CM, and FFP2 (with exhaling valve) than without a mask^12^.

However, as mentioned before, in many studies on wearing masks under physical stress, short-term loads up to 300 watts and more were carried out on bicycle ergometer^13, 14^ or only young, well-trained subjects were included^11^, not representing typical conditions in everyday life or at German workplaces. In addition, in most studies during cardiopulmonary exercise test (CPET), masks were worn under a silicone CPET mask, which has already been discussed as an influencing factor^15^ and which also did not allow blinding of the mask and non-mask situation, respectively.

Therefore, we investigated the influence of three commonly used types of masks (SM, CM, FFP2) in a normally trained cohort under different workloads corresponding to German workplaces. Another focus of our partially double-blinded study, that was recently published, was on cardiopulmonary parameters. It showed that wearing face masks at rest and under workload changed the breathing pattern in the sense of physiological compensation^16^. Overall, the data indicated that mask wearing causes no health risk for healthy subjects, but leads to enhanced breathing resistance due to the mask material in combination with increased humidity and temperature behind the mask.

While general heat perception during a 1-h walk with SM increased similar as without SM, complaints of facial warmth constituted the single most frequent complaint (52%) with SM^17^. This suggests that, especially in the case of physical exertion, it makes a difference whether the subjects were asked for perceived exertion in general (Borg scale) or for the direct impact of the mask on the mouth-nose region or for specific symptoms such as headache.

Since, in addition to physical health burden, psychological distress also plays a role in risk assessment at the workplace, the aim of the partially double-blinded randomized crossover study presented here was subjective impairment of the study participants by wearing three different mask types compared to the no mask situation and how the impairment changes with increasing physical exertion and longer wearing time. Furthermore, it should be examined whether the subjective perception of heat and humidity corresponds to the data actually measured. Since subjective impairment includes cognitive performance this was also tested under mask wearing conditions at rest. As employers have to determine a suitable mask type and tolerable periods of wearing time recommended for workers, the question arose whether the subjective feeling during mask wearing is influenced by individual factors like age, sex, atopy, or a “sensitive” disposition.

## Methods

A total of 40 subjects covering a wide age range participated in the study. They were recruited through information on the website of our institute. Exclusion criteria were absolute and relative contraindications for CPET according to the American Thoracic Society^18^. Subjects underwent a baseline examination consisting of medical history, recruitment questionnaire, physical examination, routine laboratory tests, electrocardiogram, a pulmonary function test and an initial CPET.

Specific IgE antibodies (sIgE) to ubiquitous aeroallergens (atopy screen sx1, Phadiatop) were measured with the ImmunoCAP 250 system (ThermoFisher Scientific, Phadia AB, Uppsala, Sweden). A positive atopic status was assumed in case of a sIgE concentration to sx1 ≥ 0.35 kU/L.

### Study design

The prospective, randomized, crossover study design was described in detail in a previous manuscript dealing with the influence of masks on cardiopulmonary performance^16^. All parts of the study, including recruitment, were conducted between September 2020 and July 2021.

Each subject was tested with four mask situations: no mask (NM) as reference, surgical mask (SM; Type II, MedicalCare & Serve industry^®^, Wilfried Rosbach GmbH, Willich, Germany), community mask (CM; van Laack^®^ GmbH, Mönchengladbach, Germany), and a filtering face piece (FFP2; Dräger X-plore^®^ 1920 NR D, Dräger^®^ Safety AG, Lübeck, Germany) in randomized order. The cross-over study consisted of two modules where normally worn masks (including leakage) were examined during physical exertion on a bicycle ergometer (ergometry) and under normal working conditions (workplace examination). In a third module (CPET) at identical physical exertion as in ergometry, mask material was presented to subjects in a double-blinded setting using a special mask adapter^16^. In brief, a round sample of the tested mask (or nothing for the no mask situation) was placed in a commercially available opened, empty bacterial filter and airtight closed with adhesive tape and a metal clamp. This mask adapter was then placed between the silicone CPET mask and the measuring devices (Fig. S1). The order of the three modules, which all took place at our institute, was also randomized. For physical exertion (ergometry, CPET), a maximum of two mask situations per day were tested, with a sufficiently long regeneration time in between. The workplace examinations with the four mask situations were carried out on four different days. In all modules, each session was conducted at a comparable time of day.

Individually determined load levels resulting in a minute ventilation of 10 L/min (rest), 30 L/min (exercise (E1)), 50 L/min (E2), > 60 L/min (E3) and 10 L/min (post)—each lasting six minutes—were used for the physical exertion during ergometry and CPET. According to the German Social Accident Insurance this corresponds to light (rest and post), moderate (E1), heavy (E2), and very heavy (E3) work^16^. During the 4-h workplace examination the masks were normally worn during light/moderate work in the office or laboratory.

Cardiopulmonary parameters and blood gases were measured and perceived exertion (Borg scale) was requested as described earlier^16^. Temperature and relative humidity were recorded using a climate data logger (PeakTech 5185^®^, Ahrensburg, Germany), which was fixed with adhesive tape between nose and mouth.

### Identification of “sensitive” individuals

During the baseline examination, the participants filled out questionnaires including questions on health status and specific sensitivity scales enabling a retrospective characterization of the study group in terms of identifying “sensitive” individuals. The latter had no relevance for the in- or exclusion of individuals.

One of the sensitivity scales was the Discomfort Intolerance Scale (DIS) that measures the level of agreement with statements about tolerance of discomfort^19^. Two distinct sub-factors entitled Intolerance of Discomfort or Pain (DIS-I) (2 items; e.g. “I can tolerate a great deal of physical discomfort”-reverse scored), and Avoidance of Physical Discomfort (DIS-A) (3 items; e.g. “I take extreme measures to avoid feeling physically uncomfortable”) were calculated aside from the global DIS-score. Also included were a questionnaire on environmental worry (Environmental Worry Scale (EWS), 5 items) constructed to express possible negative thoughts and associations towards negative effects and personal threats by environmental factors like for example “I often think about the fact that I am taking pollutants into my body”^20^, and the Positive and Negative Affectivity Schedule (PANAS) measuring the extent to which the subject generally experiences positive or negative emotions^21^. For the purpose of the present study, only the negative affectivity subscale (PANAS-NA) containing 10 negative items (upset, guilty, scared, hostile, irritable, ashamed, nervous, jittery, afraid, distressed) was used to assess the trait-like tendency to experience negative affect states. In addition, recruitment tools included two questionnaires on self-reported sensitivity to chemicals. The Chemical Odor Sensitivity Scale (COSS) is an 11-item scale for assessing trigeminal (e.g. shortness of breath, coughing, sickness, and nausea) and olfactory (e.g. perceived unpleasantness) mediated responses when exposed to chemicals such as paint or everyday odors like perfume^22^. The main focus of the Chemical Sensitivity Scale (CSS, 21 items) is on affective reactions and behavioral disruptions by chemicals^23^.

From these data a relative cut-off score was calculated to divide subjects into either lower or higher “sensitive” persons. Except for the EWS, the sensitivity factors were median-split: MD(DIS) ≥ 17; MD(DIS-I) ≥ 4; MD(DIS-A) ≥ 8; MD(PANAS-NA) ≥ 16; MD(COSS) ≥ 10; MD(CSS) ≥ 50. Regarding the EWS, subjects were counted as subjects with heightened environmental worry if they partly or fully agreed on at least one of the five items^24^.

### Experimental measurements and questionnaires during the study

Comfort Score and Symptom Score were assessed with mask (or without in the NM situation) before (pre) and after (post) ergometry and CPET, and Comfort Score also within the last 20 s of each load level. During the 4-h workplace examination, Comfort Score and Symptom Score were assessed 30 min before mask wearing (pre), 30, 60, 90, 120, 150, 210, and 240 min during mask wearing, as well as 30 min (post) after the end of mask wearing. Additionally, cognitive performance was assessed at the beginning and at the end of the workplace examination.

#### Comfort Score questionnaire

The Comfort Score questionnaire with ten items (humidity, heat, breathing resistance, itchiness, tightness, saltiness, feeling unfit, odor, fatigue, and overall discomfort) was used in German translation to quantify the perception of comfort/discomfort of wearing a mask. Sensations had to be rated on a 10-point rating scale with 1 representing ‘‘not at all’’, 5 representing ‘‘mildly’’ and 10 representing ‘‘strongly’’^13, 25^. The sum of the Comfort Score was the sum of all items except overall discomfort. The Comfort Score was used to determine the direct impact of the masks on the mouth-nose region and on the breathing comfort.

#### Symptom Score questionnaire

The occurrence and intensity of more general impairments caused by wearing a mask (e.g. headache, dizziness) was recorded with the Symptom Score questionnaire in German translation. The Symptom Score consists of 16 complaints and 4 dummy complaints that have been shown to be sensitive to CO_2_ inhalation effects^26^. Each of these complaints was rated on a 5-point graded scale (1 = not at all, 2 = slightly, 3 = medium, 4 = strong and 5 = very strong). The total complaints score (sum of Symptom Score) was a sum of these 16 complaints (16—80).

#### Cognitive performance

After the beginning and before the end of the 4-h workplace examination subjects performed a math and a spelling test to examine possible effects of mask wearing on cognitive performance. The duration of each test, which ran automatically on a computer, was 11 min and the spelling test was administered after the math test.

After the instruction, which was shown for 5 s, each of the 93 tasks was visible for 7 s. The elapsed time was displayed in the form of a progress bar below the task. Subjects were asked to answer as quickly and correctly as possible. In the math test, the subjects had to perform simple mental arithmetic. The tasks consisted of multiplying two numbers between 1 and 10 and subtracting a one or two-digit number from this product (e.g. (9 × 5) − 17 = 28). The subjects had to decide, if the correct calculation result was smaller, larger or equal compared with the given solution number. In the spelling test, subjects had to recognize misspelled words. These words had one or two mistakes or were correctly spelled.

If subjects did not respond within the allotted time, the next task was automatically presented, and the task was scored as error (omission). The number of correct and false answers, omissions, and the mean response time were counted.

### Statistical analysis

Data (raw scores) of Comfort and Symptom Scores are expressed as median (minimum–maximum) and interquartile range (IQR 75–25) and presented by boxplots (box: median, 25th-75th percentile; whiskers: 5–95 percentile).

A generalized linear mixed (GLM) model, along with generalized estimating equations (GEE), was used on the logarithmized sum values of Comfort Score and Symptom Score as dependent variable. The GEE procedure extends the GLM to allow for analysis of repeated measurements. Here, load level and time of measuring were included as influencing factors. This approach allows intraindividual comparison at the different examination times (i.e., each subject is compared to him/herself). Least Squares Means were calculated based on these models. The situation without mask at each load level (pre, E1, E2, E3, post) or each time of measurement (pre, 30, 60, 90, 120, 150, 210, 240, post) was used as reference. Also considered were influencing factors such as sex, age (per 10 years) and height (per 10 cm). Further influencing factors on the sum of Comfort Score and cognitive function were tested by including them individually as potential factors to the model.

Since the GLM model was only applicable to the sum of Comfort and Symptom Score, we processed the data for the single questions using analysis of variance (Friedman test, Dunn's multiple comparisons test as post-hoc test). For Comfort Score, at each load level (pre, E1, E2, E3, post) or each time of measurement (pre, 30, 60, 90, 120, 150, 210, 240, post) the data with mask were compared with the respective non-mask situation (reference).

Pearson's chi-squared test was used to examine possible differences between men and women related to the different sensitivity scales. The Spearman rank correlation was calculated to predict the monotone association between parameters for correlations. This is further visualized by contrasting the parameters in a heat map.

A p-value of < 0.05 was considered statistically significant. Analyses were performed using SAS 9.4 (SAS Institute, Cary, NC, USA). Figures were drafted with SAS 9.4 and GraphPad Prism version 9 (GraphPad Software, San Diego, CA, USA).

### Ethical approval

The Ethics Committee of the medical faculty of the Ruhr-University Bochum gave approval to perform the study (Reg. No.: 20–7024) and all subjects gave written informed consent. The person shown in Fig. S1 gave written informed consent for publication of identifying images in an open access-online publication. All methods were performed in accordance with the relevant guidelines and regulations. The study was conducted in accordance with the latest revision of the ethical standards set down by the Declaration of Helsinki.

## Results

Forty subjects (20 women, 20 men) between 19 and 65 years participated in the study. About two thirds of the participants were employees of our institute (IPA) and worked in the office or laboratory. The other subjects were external with different professions, but they all did light office/computer work at our institute during the 4-h workplace examinations. The study participants were moderately to well trained and the proportion of well-trained subjects was similar for men and women (Table 1). In general, more women than men were categorized as “sensitive” according to the different sensitivity scales.

**Table 1:** Characteristics of the study participants and numbers of “sensitive” subjects according to the different sensitivity scales.

	All	Men	Women
	N = 40	N = 20	N = 20
Age (years), median (range)	47 (19–65)	49 (19–65)	44 (23–61)
Height (cm), median (range)	180 (160–196)	185 (175–196)	170 (160–182)
Weight (kg), median (range)	75 (57–121)	85 (72–121)	68 (57–90)
Smoker, n (%)	8 (20%)	2 (10%)	6 (30%)
Former smoker, n (%)	13 (33%)	7 (35%)	6 (30%)
Mild asthma, n (%)	2 (5%)	2 (10%)	0 (0%)
Arterial hypertension, n (%)	5 (13%)	3 (15%)	2 (10%)
Atopy (sx1 ≥ 0,35 kU/L), n (%)	22 (55%)	11 (55%)	11 (55%)
“Well-trained” according to PWC_130_	20 (50%)	9 (45%)	11 (55%)
“Sensitive” according to			
EWS, n (%)	16 (40%)	7 (35%)	9 (45%)
DIS, n (%)	22 (55%)	9 (45%)	13 (65%)
DIS-I, n (%)	22 (55%)	10 (50%)	12 (60%)
DIS-A, n (%)	18 (45%)	9 (45%)	9 (45%)
PANAS-NA, n (%)	22 (55%)	9 (45%)	13 (65%)
COSS, n (%)	18 (45%)	6 (30%)	12 (60%)
CSS, n (%)	20 (50%)	8 (40%)	12 (60%)
“Sensitive” according to at least 5 sensitivity scales, n (%)	11 (28%)	3 (15%)	8 (40%)

The cut-offs of PWC_130_ (physical working capacity at a heart rate of 130 beats per minute) for trained subjects were defined as > 1.5 (men) and > 1.4 (women)^27^.

Sensitivity scales: Environmental Worry Scale (EWS), Discomfort Intolerance Scale (DIS), Intolerance of Discomfort or Pain (DIS-I), Avoidance of Physical Discomfort (DIS-A), Negative Affectivity (PANAS-NA), Chemical Odor Sensitivity Scale (COSS), Chemical Sensitivity Scale (CSS).

### Comfort and symptom score

**Figure 1:**
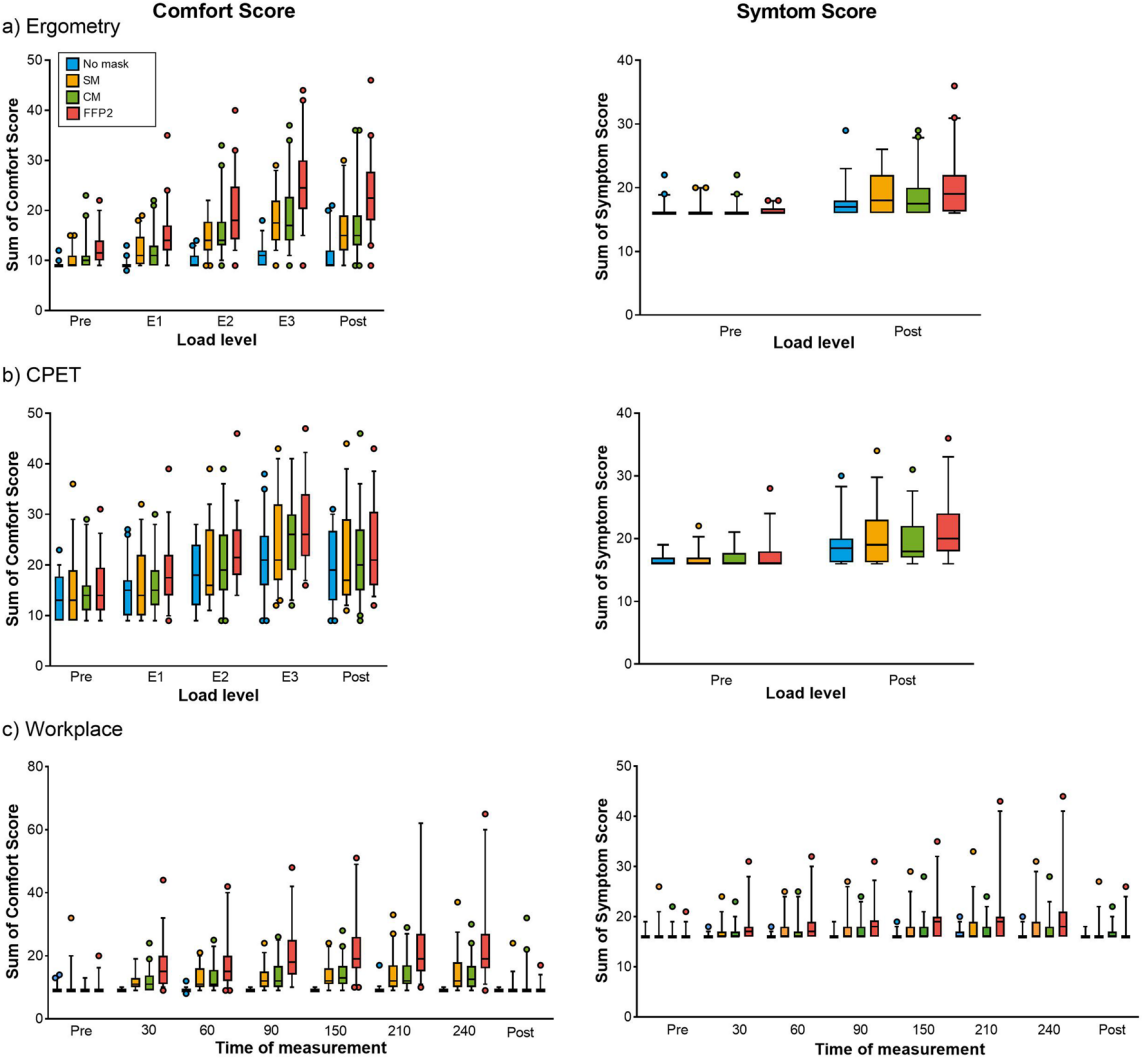
Sum of Comfort and Symptom Score during ergometry (**a**), CPET (**b**) and workplace examination (**c**) in 40 subjects wearing no mask (blue), surgical mask (yellow), community mask (green), and FFP2 mask (red). P values are shown in Table 3. During ergometry and CPET, Comfort and Symptom Score were assessed before (pre) and after (post), and Comfort Score also within the last 20 s of each load level. The load levels correspond to light work (pre and post), moderate (E1), heavy (E2) and very heavy work (E3).

The complete data for Comfort Score (mouth-nose region) and Symptom Score (more general symptoms) are displayed in Tables S1 and S2. As an example, the sums of Comfort and Symptom Score during ergometry, CPET and workplace examination are depicted as boxplots in Fig. 1.

In general, the sum values with mask, especially with FFP2, were higher than without mask at each load level (E1, E2, E3) or time of measurement. The score increased with increasing physical exertion during ergometry and CPET, even without a mask. In CPET, it must be considered that due to the fact that the subjects always wore a tight-fitting silicone CPET mask, i.e. also in the non-mask situation, impairments and symptoms were reported even without a mask (Fig. 1b).

Considering the data shown in Table S1, the comparison of the Comfort Score with mask at each exertion level in ergometry to the situation without mask, showed a significantly higher feeling of humidity, heat and difficult breathing, but also an overall discomfort with mask (Table 2a). Even in the blinded scenario (CPET), subjects felt significantly more impaired in breathing and comfort and reported more humidity and heat when wearing a FFP2 (Table 2b). After the 4-h workplace examination, the subjective impairment with mask was already significantly more pronounced after 30 min than without mask. Again, this was particularly related to humidity, heat, difficult breathing and overall discomfort, but significantly higher scores were reported for all questions except “salty” when FFP2 was worn for 60 min or longer (Table 2c).

**Table 2:**
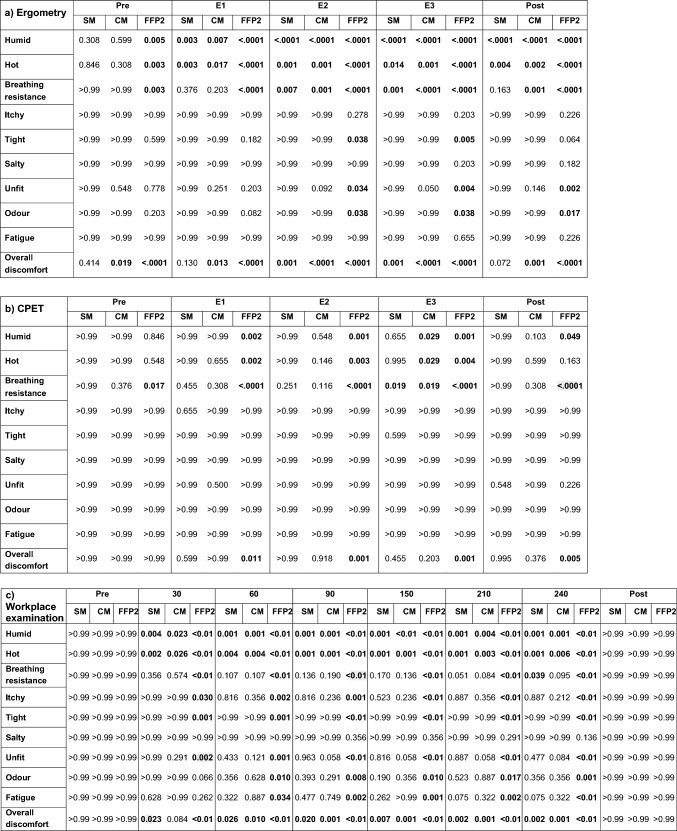
Results of statistical analysis of the data from Comfort Score questionnaire in 40 subjects during ergometry (a), CPET (b) and workplace examination (c).

For each load level (pre, E1, E2, E3, post) or time of measurement (pre, 30–240 min., post), the data with mask (SM, CM, FFP2) were compared with the respective non-mask situation (NM) using Dunn’s multiple comparisons test after Friedman test. Shown are p values (< 0.05 marked bold).

*NM* no mask, *SM* surgical mask, *CM* community mask, *FFP2* filtering face piece class 2, *CPET* Cardiopulmonary exercise test.

The load levels in ergometry and CPET corresponded to light work (pre and post), moderate (E1), heavy (E2) and very heavy work (E3).

In Table (c): < 0.01 means < 0.0001.

**Figure 2:**
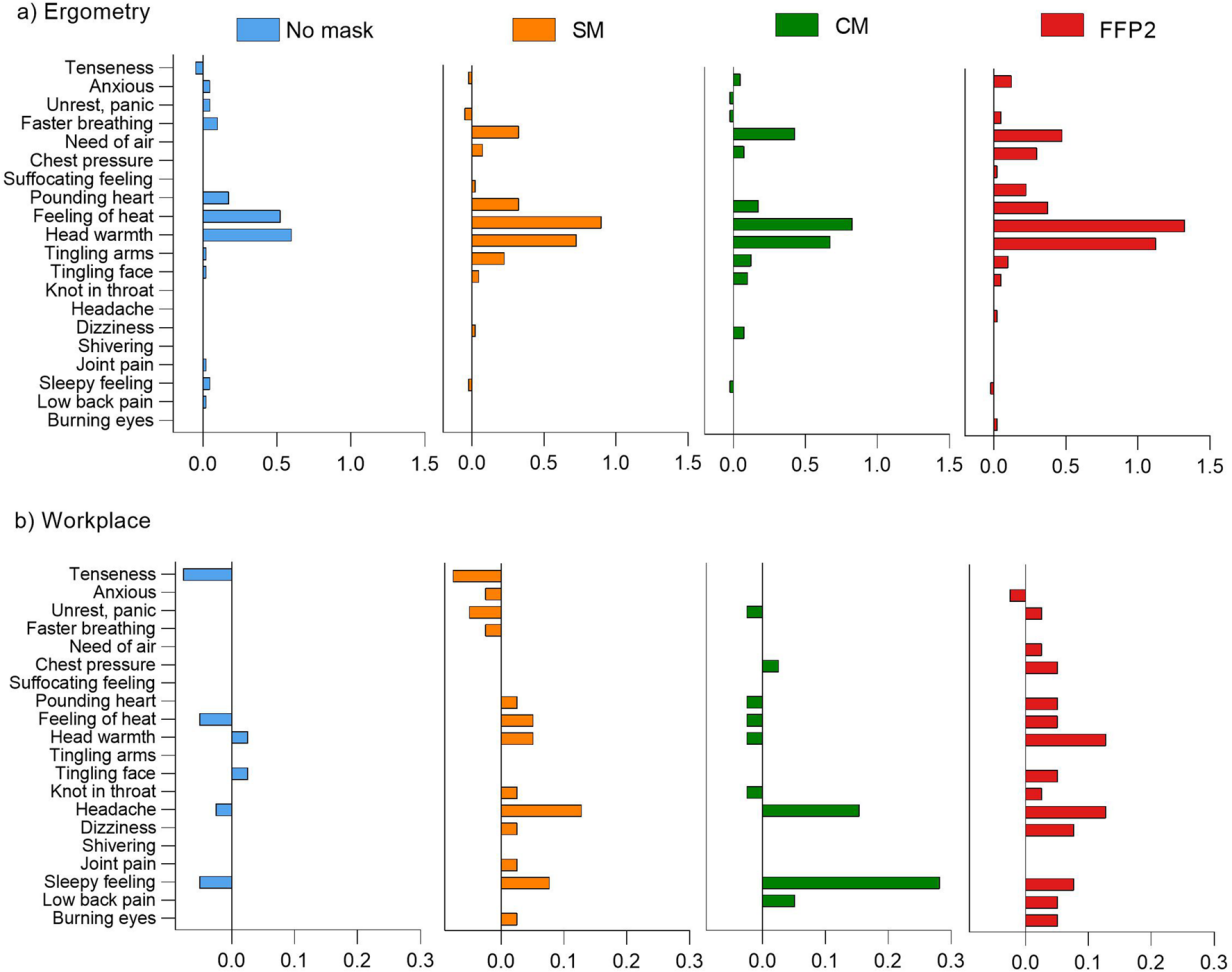
Graphical visualization of the differences in Symptom Score before and after ergometry (**a**) and the 4-h workplace measurement (**b**). For each symptom, the mean value of the 40 subjects before the examination was subtracted from the mean value after the examination. Since the Symptom Score ranged between 1 and 5, the maximum expected delta would be 4.0.

As can be seen in Table S2, rather low Symptom Scores had been reported even after physical exertion or longer wearing duration. For better visualization, the delta (∆, after minus before) for each symptom is shown in Fig. 2a and b. After ergometry, an increased feeling of heat and faster/deeper breathing were reported in particular. These changes also occurred without mask, but were more pronounced with all three mask types. The greatest difference compared to the baseline value was reported with the FFP2 (∆: 1.33 of 4.0) (Fig. 2a). Compared to ergometry, the changes in the different symptoms due to workplace examination were lower (max. ∆: 0.28 of 4.0) and in some cases even lower scores were reported after the 4-h wearing period than before (negative ∆) (Fig. 2b). However, the impairments mentioned do not only refer to the feeling of warmth, but also specific symptoms such as headache and sleepiness had been reported after wearing the masks for 4 h by some subjects.

The use of the sum of Comfort and Symptom Score enabled the analysis by means of GLM model (Table 3). With all mask types the subjects already reported significantly higher sum values of Comfort Score from the first load level (E1) or time of measurement (30 min.) than without mask, except one (CM at level E1) in ergometry and workplace examination. The strength of effects was presumably reduced (smaller ∆) in the CPET by the tight-fitting CPET mask, but also here the scores increased more with mask than without and were significantly higher with FFP2 at levels E2 and E3 (Table 3a). The effect of mask wearing on the sum of Symptom Scores was weaker. Although all three mask types caused an increase in the score (positive ∆), in most cases this was only significant for FFP2 (Table 3b).

**Table 3:** Results of generalized linear mixed (GLM) model analysis of the sum of Comfort Score (a) and Symptom Score (b) in 40 subjects without mask (NM) and with masks (SM, CM, FFP2) during physical exertion (ergometry, CPET) and during 4 h workplace examination.

	Reference	Δ mask vs. no mask		
	Sum score without mask (NM)	SM	CM	FFP2
(a) Comfort Score				
Ergometry				
Pre	9.09	0.96	1.46	3.05
E1	9.10	2.49	2.48	5.29
		**0.007**	0.077	**0.001**
E2	9.77	4.39	5.15	8.93
		** < 0.0001**	** < 0.0001**	** < 0.0001**
E3	10.77	6.57	6.94	13.70
		** < 0.0001**	** < 0.0001**	** < 0.0001**
Post	10.75	4.95	5.55	11.06
		** < 0.0001**	** < 0.0001**	** < 0.0001**
CPET				
Pre	12.89	0.53	1.03	1.62
E1	13.99	1.10	1.58	3.55
		0.576	0.636	0.097
E2	16.91	1.19	2.43	5.00
		0.677	0.389	**0.036**
E3	20.20	2.27	3.93	6.68
		0.291	0.108	**0.009**
Post	18.06	1.42	2.07	3.91
		0.569	0.610	0.218
Workplace				
Pre	9.30	0.65	0.13	0.23
30	9.04	2.49	2.53	6.53
		**0.002**	** < 0.0001**	** < 0.0001**
60	9.05	3.33	3.44	7.15
		** < 0.0001**	** < 0.0001**	** < 0.0001**
90	9.03	3.52	3.96	9.15
		** < 0.0001**	** < 0.0001**	** < 0.0001**
150	9.06	3.77	4.01	10.55
		** < 0.0001**	** < 0.0001**	** < 0.0001**
210	9.16	3.92	6.94	11.03
		** < 0.0001**	** < 0.0001**	** < 0.0001**
240	9.03	4.40	4.18	11.91
		** < 0.0001**	** < 0.0001**	** < 0.0001**
Post	9.05	0.56	0.82	0.45
		0.877	0.125	0.612
(b) Symptom Score				
Ergometry				
Pre	16.24	0.11	0.15	0.05
Post	17.62	1.17	1.00	2.41
		**0.023**	0.064	** < 0.0001**
CPET				
Pre	16.52	0.12	0.49	0.7
Post	18.74	1.07	0.72	2.08
		0.138	0.796	0.054
Workplace				
Pre	16.27	0.27	0.19	0.07
30	6.18	0.62	0.45	1.38
		0.286	0.410	** < 0.0001**
60	16.20	0.97	0.77	1.92
		0.083	0.144	** < 0.0001**
90	16.22	1.13	0.84	2.17
		**0.031**	0.097	** < 0.0001**
150	16.17	1.19	0.87	2.76
		**0.044**	0.133	** < 0.0001**
210	16.40	1.30	0.81	3.17
		**0.043**	0.211	** < 0.0001**
240	16.37	1.26	0.89	2.99
		0.057	0.174	** < 0.0001**
Post	16.18	0.47	0.34	0.73
		0.508	0.613	**0.026**

The situation without mask at each time of measurement was always used as reference. For NM geometric mean, for the three mask types (SM, CM, FFP2) differences to NM (Δ) and p values (< 0.05 marked bold) are shown.

The load levels correspond to light work (pre and post), moderate (E1), heavy (E2) and very heavy work (E3).

*NM* no mask, *SM* surgical mask, *CM* community mask, *FFP2* filtering face piece class 2, *CPET* cardiopulmonary exercise test.

The evaluation of influencing factors (age, sex, atopy, smoking, training status (well-trained or not), and “sensitivity” according to the seven sensitivity scales) on the sum of Comfort Score resulted in the observation that “sensitivity” according to the DIS sensitivity scale was significantly associated with higher sum values of Comfort Score (stronger impairment) during ergometry (OR 1.14, 95% CI 1.02–1.27, p = 0.018). During workplace examination, women showed significantly lower Comfort Scores than men (OR 0.84, 95% CI 0.72–0.99, p = 0.043) and also with increasing age (per 10 years) lower Comfort Scores were reported (OR 0.95, 95% CI 0.92–0.98, p = 0.001). In contrast to this, atopic subjects had significantly higher scores than non-atopics (OR 1.16, 1.06–1.27, p = 0.002). However, those atopics suffering from allergic symptoms (n = 10) did not show significantly higher Comfort Scores than atopics without allergic symptoms (n = 12) (p = 0.27 workplace examination; p = 0.43 ergometry).

### Correlations

**Figure 3:**
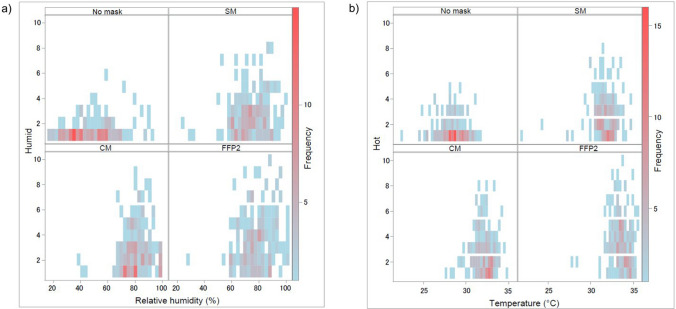
Graphical presentation of the agreement between the measured median values of humidity (**a**) and temperature (**b**) under the mask and without mask and the values for “Humid” and “Hot” of the Comfort Score questionnaire. Data of 40 subjects across all levels of ergometry were considered. *NM* no mask, *SM* surgical mask, *CM* community mask, *FFP2* filtering face piece class 2.

Figure 3 depicts the correlation of humidity and temperature measured during ergometry between mouth and nose with or without mask (data see^16^) with the corresponding information from Comfort Score questionnaire. Humidity and temperature were higher under all three mask types than without mask. Both the highest median humidity and temperature were measured behind the FFP2. In good agreement with this, scores for "humid" and "hot" up to the maximum of 10 were reported exclusively with FFP2.

**Figure 4:**
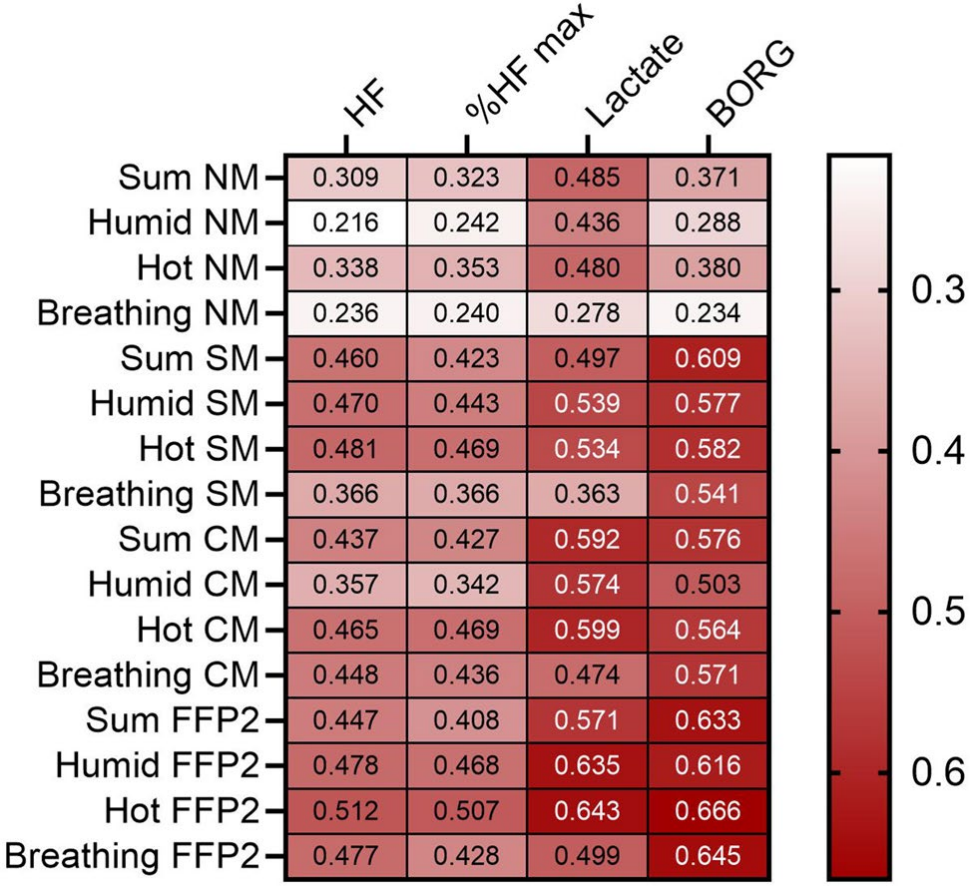
Heatmap of the correlation between physiological parameters (heart frequency (HF), percent from maximum HF, lactate) and perceived physical exertion (Borg scale) with selected items and the sum of the Comfort Score questionnaire. Data of 40 subjects across all levels of ergometry were considered. *NM* no mask, *SM* surgical mask, *CM* community mask, *FFP2* filtering face piece class 2.

Selected data from the Comfort Score were positively correlated with physiological parameters (e.g. heart frequency (HF), lactate) during ergometry (Fig. 4). For the situation with mask, especially FFP2, there was a moderate to strong correlation especially between the items "humid" and "hot" of Comfort Score with lactate and Borg scale, respectively.

### Cognitive performance

Math and spelling tests were carried out before and after 4-h workplace examination investigating the influence of mask wearing on cognitive performance. The proportions of correct (false) answers and omissions were comparable with and without mask, and even after the 4 h mask wearing, the numbers of errors (false answers and omissions) did not increase (Table 4). On the contrary, in the math test, the number of errors was lower in the second measurement both with and without a mask. The reaction time in the math test was consistently higher than in the spelling test. Overall, concerning cognitive performance, no significant differences could be detected between the situation with and without mask. Statistical analyses did not yield significant results, even not when testing for influencing factors on the cognitive performance.

**Table 4:** Cognitive performance: results of math and spelling tests before and after the 4-h workplace examination in 39 subjects without mask (NM) and with three different mask types.

Mask type	Math test before	Math test after	Spelling test before	Spelling test after
Correct answers, n (%)				
NM	2683 (74%)	2851 (79%)	3046 (82%)	2835 (80%)
SM	2520 (69%)	2623 (72%)	2914 (80%)	2925 (81%)
CM	2589 (71%)	2729 (75%)	2913 (80%)	2923 (81%)
FFP2	2525 (70%)	2723 (75%)	2933 (81%)	2907 (80%)
False answers, n (%)				
NM	735 (20%)	622 (17%)	646 (18%)	675 (19%)
SM	855 (24%)	804 (22%)	662 (18%)	677 (19%)
CM	784 (22%)	705 (19%)	674 (19%)	684 (19%)
FFP2	792 (22%)	735 (20%)	653 (18%)	695 (19%)
Omissions, n (%)				
NM	209 (6%)	154 (4%)	28 (1%)	24 (1%)
SM	252 (7%)	200 (6%)	51 (1%)	25 (1%)
CM	254 (7%)	193 (5%)	40 (1%)	20 (1%)
FFP2	310 (9%)	169 (5%)	41 (1%)	25 (1%)
Response time for correct answers [ms], mean ± SD				
NM	4510 ± 1339	4327 ± 1356	3833 ± 1233	3745 ± 1243
SM	4603 ± 1361	4483 ± 1372	3898 ± 1268	3765 ± 1260
CM	4532 ± 1394	4371 ± 1432	3855 ± 1239	3711 ± 1239
FFP2	4514 ± 1408	4320 ± 1401	3796 ± 1258	3662 ± 1231
Response time for false answers [ms], mean ± SD				
NM	5427 ± 1238	5251 ± 1277	4345 ± 1359	4253 ± 1355
SM	5453 ± 1240	5229 ± 1379	4529 ± 1348	4301 ± 1427
CM	5294 ± 1377	5088 ± 1449	4314 ± 1354	4258 ± 1384
FFP2	5278 ± 1501	5087 ± 1510	4382 ± 1326	4209 ± 1343

The total numbers of tasks were 3,627 with each type of mask in each test (math and spelling).

*NM* no mask, *SM* surgical mask, *CM* community mask, *FFP2* filtering face piece class 2.

## Discussion

According to our partially double-blinded, randomized, cross-over study, wearing SM, CM or especially FFP2 results in subjective discomfort, particularly including difficult breathing and an uncomfortable feeling of humidity and heat underneath the mask. The feeling of discomfort increases with higher physical exertion and to a lesser extent with longer wearing time. Also, in the blinded CPET scenario, the subjects report significantly stronger impairment with FFP2 than without mask. Symptoms not directly affecting the mouth-nose region are reported rather rarely. However, headache and sleepiness occurred occasionally after 4-h workplace examination. No changes in cognitive performance are found as a result of wearing a mask.

Although some authors have already reported subjective sensations of wearing a mask, most studies solely surveyed subjects’ perceived exertion using the Borg scale. In studies using additional instruments, subjects were interviewed either at rest^28^, or at moderate^17^ or maximum exertion^13^. In contrast, in our study all subjects underwent both exercise ergometry and 4-h workplace examination, and in addition to the subjects' perceived exertion, two specific and proven questionnaires, the Comfort and Symptom Score questionnaires, were administered.

As a particular strength of our study, we conducted a subjective assessment of mask wearing during physical exertion in a double-blinded manner (CPET). Even though this did not correspond to normal mask wearing because of the tight-fitting CPET mask, it confirmed the main outcomes of ergometry. Blinding seems to be particularly important in the case of subjective assessments as it is well known that blinding makes it difficult to bias results intentionally or unintentionally and so helps ensure the credibility of study conclusions^29, 30^. To our knowledge, blinding was only attempted in one previous study^14^, where subjects' perceived exertion was recorded using the Borg scale during physical exertion with masks (SM and FFP2). For single blinding of the no mask situation, a large piece of material was cut out centrally from a SM and worn underneath the silicone CPET mask. The actual mask testing (SM and FFP2) could not be blinded this way. However, also in the study of Mapelli and co-workers^14^, at peak exercise, all subjects revealed progressively higher perceived exertion values from no mask to SM to FFP2. In addition, the GLM model-based analyses used in our study allowed an intraindividual comparison at the different examination times (i.e. each subject is compared to him/herself) and a consideration of repeated measurements.

Another advantage of our study is the measurement of temperature and humidity underneath the mask as well as the recording of physiological parameters^16^ from the same 40 subjects in parallel to their subjective sensation when wearing masks. Thus, we were able to look for associations between subjective and physical/ physiological data.

### Comfort Score

The finding that in particular the feeling of humidity, heat and difficult breathing, but also the overall discomfort is increased when wearing a mask, especially FFP2, is in good agreement with the results of a previous study where the Comfort Score questionnaire has been used in ten subjects wearing different mask types for 100 min during intermittent exercise on a treadmill^25^. Also, Fikenzer et al.^13^ used this questionnaire to study the comfort/discomfort of wearing a mask in 12 male subjects during maximum exercise in CPET. In agreement to our finding, the masks lead to severe subjective discomfort during exercise and FFP2/N95 were perceived more uncomfortable than SM. Fikenzer and coworkers^13^ reported that breathing resistance, heat, tightness and overall discomfort were the items with the strongest influence on subjective feeling. However, since they had tested the masks under a tight-fitting CPET mask, this may explain the strong feeling of tightness that we did not observe. This illustrates the benefit of performing CPET (to observe the breathing pattern and as a blinded scenario) and ergometry (identical load levels with normal mask wearing) in parallel.

Humidity and temperature were highest under the FFP2 and showed a good concordance with the subjective perception of humidity and warmth underneath the mask in our study. Other authors also measured the temperature and humidity under the mask or asked about associated complaints, but only a few studies did both.

Liu and coworkers^28^ examined 12 male students wearing different masks for about 100 min at rest. Thermal imaging test results were consistent with the subjective thermal and wet sensation and wearing a KN95 respirator provided the strongest discomfort^28^.

Scarano et al.^31^ investigated 20 subjects while wearing a SM on the first day and a N95 respirator on the second day, each for 1 h. Using infrared imaging, significantly lower temperature changes were detected with N95 during the breathing act and after the mask removal a significantly higher perioral facial temperature was observed compared to SM (p < 0.05). In accordance with the infrared imaging the subjective feeling of humidity, heat, breathing difficulty, and discomfort was significantly higher for N95 (p < 0.01). Subjects wearing the N95 touched it 25 times to move it, while those wearing the SM performed this gesture 8 times. The authors concluded that it is better to wear a SM correctly than a respirator (N95, FFP2) leading to temporary withdrawal of the mask from the face due to the discomfort^31^.

Moderate to strong correlations were observed between the perceived exertion of the subjects in general (Borg score) and the specific feelings asked in the Comfort Score questionnaire. In the case of physical exertion with FFP2, Comfort Scores correlated well with the Borg score and the physiological parameters for physical exertion (HF, lactate). This might be in line with the finding of another study that especially less trained people (strong increase in HF) seem to experience symptoms such as dyspnea or feeling hot, especially with FFP2, sometimes even at low levels of exertion^32^. However, the training status as an influencing factor on the sum of Comfort Scale did not show any significant result in our study. This may be caused by the inaccurate estimation of the training status (well-trained or not) using the PWC_130_.

Subjects who were categorized as being more intolerant of discomfort according the DIS sensitivity scale, perceived more discomfort during ergometry, whereas male and atopic subjects perceived more discomfort during workplace measurements.

Since discomfort intolerance was found to influence reporting of acute health effects in response to a biological stressor in healthy subjects^33^, it seems plausible that individuals with an elevated DIS score also report higher Comfort Scores. The higher Comfort Scores in atopics can also be explained in view of the discussion about whether atopics react more strongly to various stimuli. However, the investigation of the influence of atopy on the irritative effects of ethyl acrylate on 22 subjects, showed that atopic subjects did not report higher eye or nose irritation intensity ratings than non-atopics^34^. While in a study on 104 subjects wearing an N95 respirator during standardized simulated work tasks, female sex was associated with stronger effects on several self-reported scales^35^, this was exactly the opposite in our study, where male subjects experienced more discomfort during workplace examination. In several studies women reported symptoms more frequently than men^36^. However, this was often associated with psychosocial work stress and poorer working conditions for women, and presumably these factors did not play a role in our study population.

### Symptom Score

Symptoms that did not directly affect the mouth-nose region and the breathing comfort were mentioned rather rarely, but headache and sleepiness were reported by some study participants at the end of the 4-h workplace examination. Headaches was a prominent symptom reported by 81% of 158 healthcare workers of high-risk hospital areas while wearing N95 respirators for an average of 6 h per day during the SARS-CoV-2 pandemic^37^. In a study conducted during the Severe Acute Respiratory Syndrome (SARS) outbreak, 37% of 212 healthcare workers reported N95 face mask-associated headaches^38^. Both studies showed that headaches were most likely to occur in subjects who wore a mask for more than 4 h and had pre-existing headaches. Pre-existing headaches were not reported by study participants of our study. This could explain why the subjects wearing masks for 4 h in our study suffered only rarely and rather weakly from headaches. In addition, our workplace examination represented light/moderate work and the participants were aware that it was a short-term study situation, hardly comparable to the daily physical and mental stress of healthcare workers in high-risk hospital areas. It can be assumed that long lasting and mental stress and strain conditions further promote the development of headaches.

### Cognitive performance

An additional potential impairment from wearing masks concerns cognitive performance, which is why a math and a spelling test was carried out to simulate office work. No negative impact of mask wearing on the cognitive performance was detectable in accordance with other studies^10, 39^. Also, in a study on 133 children aged 11–14 years, the wearing of face masks (SM or FFP2) for 90 min had no significant influence on concentration and cognition^40^.

Our finding that the number of errors in the math test was lower in the second measurement both with and without mask can probably be attributed to a training effect. The consistently longer reaction time in the math test compared to the spelling test is probably due to a higher complexity of the tasks. In agreement with Haber et al.^35^ who could not demonstrate gender-specific differences in the ability to concentrate of 104 volunteers with and without N95, no factors influencing cognitive performance in connection with mask wearing could be identified in our study.

### Limitations and future research

Although different mask types and exercise intensities (load levels) have been tested in this study, the influence of ambient temperature was not considered. It is now known that the subjective perception of heat and humidity underneath the mask depends on the ambient temperature^41^ and that these effects are amplified during high-intensity interval exercise in hot environments^42^.

Although some subjects with mild asthma were included in our collective, a subgroup analysis of e.g. asthmatics was not possible. The physiological and subjective effects of wearing masks on subjects with respiratory diseases like asthma or chronic obstructive pulmonary disease (COPD) should be further elucidated in studies to come. One former study reported significant interactions in such manner that disease status modified the effect of respirator type^35^. According to this, asthmatics may be more prone to detecting inspiratory loads and to panic type response than healthy subjects or patients with COPD, who have chronically increased airflow resistance.

## Conclusion

Wearing a mask had no effect on cognitive performance during light to moderate work, but resulted in subjective discomfort, in particular difficult breathing and the feeling of humidity and heat under the mask. Feeling of discomfort increased with progressing time of mask wearing and rising physical exertion. Effects correlated well with physiological parameters and with temperature and humidity underneath the mask. Validated by double-blinded testing, it was shown that subjective impairment was similar with SM and CM and most pronounced with FFP2. Subjects with a higher index for intolerance and avoidance of discomfort were generally more likely to report increased discomfort when wearing a mask during physical exertion. Thus, consideration of individual factors and expert advice are important when determining protective measures such as the type of mask to be used and the duration of mask wearing as part of the risk assessment.

## Supplementary Information

Supplementary Tables.

Supplementary Figure S1.

Supplementary Legends.

## Data Availability

The datasets generated during and/or analyzed during the current study are available from the corresponding author on reasonable request.
