# Supplementary material for: Influence of face masks on the subjective impairment at different physical workloads
Source: Sci Rep. 2023 May 19;13:8133. doi: 10.1038/s41598-023-34319-0 (PMC10196290; doi:10.1038/s41598-023-34319-0)
Supplement: Supplementary file 1 — Supplementary Tables. [file 41598_2023_34319_MOESM1_ESM.docx]

Table S1: Results of Comfort Score questionnaire of 40 subjects without mask (NM) and with three different mask types (SM, CM, FFP2) at different load levels (pre, E1, E2, E3, post) during ergometry (a), CPET (b), and at different timepoints (pre, 30-240 min., post) during workplace examination (c).

| **a) Ergometry** | **Pre** | | | | **E1** | | | | **E2** | | | | **E3** | | | | **Post** | | | |
| --- | --- | --- | --- | --- | --- | --- | --- | --- | --- | --- | --- | --- | --- | --- | --- | --- | --- | --- | --- | --- |
|  | **NM** | **SM** | **CM** | **FFP2** | **NM** | **SM** | **CM** | **FFP2** | **NM** | **SM** | **CM** | **FFP2** | **NM** | **SM** | **CM** | **FFP2** | **NM** | **SM** | **CM** | **FFP2** |
| **Humid**  Median  IQR 75-25  Min-Max | 1  0  1-1 | 1  1  1-3 | 1  0  1-5 | 1  1  1-4 | 1  0  1-2 | 2  1  1-4 | 2  1  1-5 | 2  2  1-5 | 1  0  1-2 | 3  1  1-5 | 3  1  1-6 | 4  3  1-7 | 1  1  1-4 | 3  2  1-8 | 4  3  1-8 | 5  3  1-9 | 1  1  1-6 | 3  3  1-8 | 3  3  1-9 | 5  3  1-10 |
| **Hot**  Median  IQR 75-25  Min-Max | 1  0  1-3 | 1  1  1-3 | 1  1  1-4 | 2  1  1-4 | 1  0  1-3 | 2  1  1-4 | 2  1  1-4 | 2  1  1-5 | 1  1  1-4 | 3  1  1-5 | 3  2  1-6 | 4  2  1-7 | 2  2  1-5 | 3  2  1-8 | 4  2  1-8 | 5  2  1-9 | 1  2  1-10 | 3  2  1-7 | 3  3  1-8 | 4  3  1-10 |
| **Breathing resistance**  Median  IQR 75-25  Min-Max | 1  0  1-2 | 1  0  1-2 | 1  0  1-3 | 2  1  1-4 | 1  0  1-2 | 1  1  1-3 | 1  1  1-3 | 2  1  1-5 | 1  0  1-2 | 2  1  1-5 | 2  2  1-6 | 3  1  1-7 | 1  0  1-4 | 2  2  1-7 | 3  2  1-6 | 5  4  1-9 | 1  0  1-3 | 1  1  1-6 | 2  1  1-6 | 3  2  1-7 |
| **Itchy**  Median  IQR 75-25  Min-Max | 1  0  1-1 | 1  0  1-2 | 1  0  1-2 | 1  0  1-3 | 1  0  1-1 | 1  0  1-2 | 1  0  1-2 | 1  0  1-2 | 1  0  1-1 | 1  0  1-2 | 1  0  1-3 | 1  1  1-4 | 1  0  1-1 | 1  0  1-3 | 1  0  1-4 | 1  1  1-4 | 1  0  1-1 | 1  0  1-2 | 1  0  1-5 | 1  1  1-3 |
| **Tight**  Median  IQR 75-25  Min-Max | 1  0  1-1 | 1  0  1-1 | 1  0  1-2 | 1  0  1-4 | 1  0  1-1 | 1  0  1-2 | 1  0  1-2 | 1  1  1-5 | 1  0  1-1 | 1  0  1-3 | 1  0  1-4 | 1  1  1-5 | 1  0  1-1 | 1  0  1-4 | 1  0  1-4 | 1  2  1-5 | 1  0  1-8 | 1  0  1-2 | 1  0  1-4 | 1  1  1-6 |
| **Salty**  Median  IQR 75-25  Min-Max | 1  0  1-1 | 1  0  1-2 | 1  0  1-2 | 1  0  1-2 | 1  0  1-1 | 1  0  1-2 | 1  0  1-2 | 1  0  1-2 | 1  0  1-1 | 1  0  1-2 | 1  0  1-2 | 1  0  1-5 | 1  0  1-2 | 1  0  1-9 | 1  0  1-3 | 1  1  1-6 | 1  0  1-3 | 1  0  1-5 | 1  0  1-3 | 1  1  1-5 |
| **Unfit**  Median  IQR 75-25  Min-Max | 1  0  1-1 | 1  0  1-3 | 1  0  1-8 | 1  0  1-5 | 1  0  1-1 | 1  0  1-3 | 1  1  1-7 | 1  1  1-5 | 1  0  1-1 | 1  0  1-4 | 1  1  1-7 | 1  1  1-5 | 1  0  1-1 | 1  0  1-4 | 1  2  1-7 | 1  2  1-6 | 1  0  1-1 | 1  0  1-3 | 1  1  1-7 | 1  2  1-7 |
| **Odour**  Median  IQR 75-25  Min-Max | 1  0  1-1 | 1  0  1-2 | 1  0  1-3 | 1  1  1-5 | 1  0  1-1 | 1  0  1-5 | 1  0  1-3 | 1  1  1-7 | 1  0  1-1 | 1  0  1-8 | 1  0  1-4 | 1  1  1-7 | 1  0  1-1 | 1  0  1-8 | 1  0  1-4 | 1  1  1-7 | 1  0  1-1 | 1  0  1-5 | 1  0  1-4 | 1  1  1-7 |
| **Fatigue**  Median  IQR 75-25  Min-Max | 1  0  1-2 | 1  0  1-2 | 1  0  1-1 | 1  0  1-3 | 1  0  1-2 | 1  0  1-2 | 1  0  1-1 | 1  0  1-2 | 1  0  1-2 | 1  0  1-2 | 1  0  1-3 | 1  0  1-4 | 1  0  1-2 | 1  0  1-3 | 1  0  1-3 | 1  1  1-3 | 1  0  1-2 | 1  0  1-2 | 1  0  1-3 | 1  1  1-4 |
| **Overall discomfort** Median  IQR 75-25  Min-Max | 1  0  1-1 | 1  1  1-4 | 1  1  1-7 | 2  2  1-5 | 1  0  1-2 | 1  1  1-5 | 1  2  1-7 | 3  1  1-6 | 1  0  1-2 | 2  2  1-5 | 2  1  1-7 | 4  2  1-6 | 1  0  1-4 | 3  2  1-6 | 3  2  1-7 | 5  3  1-9 | 1  0  1-3 | 2  2  1-6 | 2  1  1-7 | 4  3  1-7 |
| **Sum***  Median  IQR 75-25  Min-Max | 9  0  9-12 | 9  2  9-15 | 10  2  9-23 | 12  4  9-22 | 9  0  8-13 | 11  5  9-19 | 11  4  9-22 | 14  5  9-35 | 9  2  9-14 | 14  6  9-22 | 14  5  9-33 | 18  10  9-40 | 11  3  9-18 | 18  8  9-29 | 17  9  9-37 | 25  10  9-44 | 9  3  9-21 | 15  7  9-30 | 15  6  9-36 | 23  10  9-46 |

| **b) CPET** | **Pre** | | | | **E1** | | | | **E2** | | | | **E3** | | | | **Post** | | | |
| --- | --- | --- | --- | --- | --- | --- | --- | --- | --- | --- | --- | --- | --- | --- | --- | --- | --- | --- | --- | --- |
|  | **NM** | **SM** | **CM** | **FFP2** | **NM** | **SM** | **CM** | **FFP2** | **NM** | **SM** | **CM** | **FFP2** | **NM** | **SM** | **CM** | **FFP2** | **NM** | **SM** | **CM** | **FFP2** |
| **Humid**  Median  IQR 75-25  Min-Max | 1  1  1-4 | 1  1  1-4 | 1  1  1-3 | 2  1  1-4 | 2  2  1-5 | 2  2  1-5 | 2  1  1-4 | 3  1  1-6 | 3  2  1-8 | 3  2  2-6 | 3  2  1-7 | 4  2  2-8 | 4  2  1-10 | 4  3  2-10 | 5  3  1-10 | 5  3  2-10 | 4  2  1-10 | 3  3  2-10 | 4  2  1-10 | 4  3  2-10 |
| **Hot**  Median  IQR 75-25  Min-Max | 1  1  1-4 | 1  1  1-5 | 2  1  1-4 | 2  1  1-5 | 2  2  1-6 | 2  1  1-5 | 2  1  1-5 | 2  1  1-6 | 3  2  1-8 | 3  2  1-6 | 3  3  1-9 | 4  1  2-8 | 4  2  1-8 | 4  3  2-10 | 5  2  1-10 | 5  2  2-10 | 3  3  1-8 | 3  3  2-10 | 3  2  1-10 | 4  2  1-9 |
| **Breathing resistance**  Median  IQR 75-25  Min-Max | 1  1  1-4 | 2  2  1-6 | 2  2  1-5 | 2  3  1-8 | 2  2  1-6 | 2  3  1-8 | 2  1  1-5 | 3  2  1-10 | 2  3  1-6 | 3  2  1-8 | 3  3  1-6 | 4  3  2-10 | 3  3  1-7 | 4  4  1-9 | 4  3  1-10 | 6  2  3-10 | 2  2  1-6 | 2  2  1-7 | 2  2  1-6 | 4  3  1-10 |
| **Itchy**  Median  IQR 75-25  Min-Max | 1  0  1-3 | 1  0  1-4 | 1  0  1-3 | 1  0  1-2 | 1  0  1-2 | 1  1  1-4 | 1  0  1-3 | 1  0  1-3 | 1  0  1-4 | 1  1  1-5 | 1  1  1-5 | 1  1  1-3 | 1  1  1-6 | 1  1  1-7 | 1  0  1-4 | 1  1  1-8 | 1  0  1-4 | 1  0  1-6 | 1  0  1-2 | 1  0  1-5 |
| **Tight**  Median  IQR 75-25  Min-Max | 2  2  1-5 | 1  2  1-8 | 2  2  1-6 | 1  2  1-6 | 1  2  1-5 | 2  2  1-7 | 2  2  1-6 | 2  2  1-6 | 2  2  1-6 | 2  2  1-7 | 2  2  1-6 | 2  2  1-6 | 2  2  1-6 | 2  3  1-8 | 2  3  1-8 | 2  2  1-7 | 2  2  1-5 | 2  2  1-7 | 2  2  1-7 | 2  2  1-6 |
| **Salty**  Median  IQR 75-25  Min-Max | 1  0  1-1 | 1  0  1-4 | 1  0  1-6 | 1  0  1-5 | 1  0  1-3 | 1  0  1-2 | 1  0  1-2 | 1  0  1-5 | 1  0  1-3 | 1  0  1-3 | 1  0  1-3 | 1  0  1-5 | 1  0  1-4 | 1  0  1-4 | 1  0  1-3 | 1  0  1-6 | 1  0  1-6 | 1  0  1-5 | 1  1  1-6 | 1  0  1-6 |
| **Unfit**  Median  IQR 75-25  Min-Max | 1  1  1-5 | 2  1  1-6 | 1  2  1-7 | 1  1  1-6 | 1  1  1-5 | 2  2  1-6 | 2  2  1-7 | 1  2  1-6 | 2  2  1-6 | 2  2  1-6 | 2  2  1-7 | 2  3  1-6 | 2  2  1-5 | 2  2  1-6 | 2  2  1-7 | 2  3  1-7 | 1  2  1-5 | 2  2  1-6 | 2  2  1-7 | 2  2  1-6 |
| **Odour**  Median  IQR 75-25  Min-Max | 1  1  1-6 | 1  0  1-6 | 1  1  1-8 | 1  1  1-4 | 1  1  1-6 | 1  0  1-6 | 1  1  1-8 | 1  1  1-4 | 1  1  1-5 | 1  0  1-6 | 1  1  1-8 | 1  1  1-5 | 1  1  1-5 | 1  0  1-6 | 1  1  1-8 | 1  1  1-5 | 1  1  1-5 | 1  1  1-6 | 1  1  1-8 | 1  0  1-4 |
| **Fatigue**  Median  IQR 75-25  Min-Max | 1  0  1-3 | 1  0  1-4 | 1  0  1-2 | 1  0  1-3 | 1  0  1-3 | 1  0  1-3 | 1  0  1-3 | 1  0  1-4 | 1  0  1-4 | 1  0  1-3 | 1  0  1-4 | 1  1  1-4 | 1  0  1-3 | 1  0  1-4 | 1  2  1-4 | 1  2  1-5 | 1  0  1-3 | 1  0  1-4 | 1  1  1-6 | 1  1  1-4 |
| **Overall discomfort** Median  IQR 75-25  Min-Max | 3  2  1-7 | 3  2  1-6 | 3  2  1-5 | 3  2  1-7 | 3  2  1-7 | 3  2  1-7 | 3  2  1-6 | 4  2  1-8 | 3  2  1-7 | 4  3  1-7 | 4  3  1-7 | 4  2  2-10 | 4  2  1-8 | 4  3  1-8 | 4  2  1-10 | 5  2  2-10 | 3  3  1-6 | 3  3  1-7 | 4  3  1-8 | 4  2  2-10 |
| **Sum***  Median  IQR 75-25  Min-Max | 13  9  9-23 | 13  10  9-36 | 14  5  9-29 | 14  8  9-31 | 15  7  9-27 | 14  12  9-32 | 15  7  9-30 | 18  8  9-39 | 18  12  9-28 | 16  13  11-39 | 19  11  9-39 | 22  9  14-46 | 21  10  9-38 | 21  15  12-43 | 26  11  12-41 | 26  12  16-47 | 19  14  9-31 | 17  15  11-44 | 20  12  9-46 | 21  14  12-43 |

| **c)  Workplace examination** | Pre | | | | 30 | | | | 60 | | | | 90 | | | | 150 | | | | 210 | | | | 240 | | | | Post | | | |
| --- | --- | --- | --- | --- | --- | --- | --- | --- | --- | --- | --- | --- | --- | --- | --- | --- | --- | --- | --- | --- | --- | --- | --- | --- | --- | --- | --- | --- | --- | --- | --- | --- |
|  | **NM** | **SM** | **CM** | **FFP** | **NM** | **SM** | **CM** | **FFP** | **NM** | **SM** | **CM** | **FFP** | **NM** | **SM** | **CM** | **FFP** | **NM** | **SM** | **CM** | **FFP** | **NM** | **SM** | **CM** | **FFP** | **NM** | **SM** | **CM** | **FFP** | **NM** | **SM** | **CM** | **FFP** |
| **Humid**  Median  IQR 75-25  Min-Max | 1  0  1-2 | 1  0  1-4 | 1  0  1-2 | 1  0  1-3 | 1  0  1-2 | 2  1  1-4 | 2  1  1-4 | 2  2  1-5 | 1  0  1-2 | 2  1  1-4 | 2  1  1-6 | 2  2  1-5 | 1  0  1-2 | 2  1  1-5 | 2  2  1-6 | 2  1  1-6 | 1  0  1-2 | 2  1  1-5 | 2  2  1-8 | 2  2  1-7 | 1  0  1-2 | 2  2  1-5 | 2  1  1-5 | 3  2  1-7 | 1  0  1-2 | 2  1  1-5 | 2  1  1-6 | 2  2  1-8 | 1  0  1-2 | 1  0  1-2 | 1  0  1-6 | 1  0  1-3 |
| **Hot**  Median  IQR 75-25  Min-Max | 1  0  1-3 | 1  0  1-7 | 1  0  1-4 | 1  0  1-4 | 1  0  1-1 | 2  1  1-3 | 2  1  1-4 | 2  2  1-5 | 1  0  1-2 | 2  1  1-4 | 2  1  1-6 | 2  2  1-6 | 1  0  1-2 | 2  1  1-4 | 2  1  1-6 | 2  1  1-6 | 1  0  1-2 | 2  1  1-5 | 2  1  1-7 | 2  1  1-8 | 1  0  1-2 | 2  1  1-5 | 2  1  1-5 | 3  2  1-9 | 1  0  1-2 | 2  1  1-5 | 2  1  1-5 | 2  2  1-10 | 1  0  1-2 | 1  0  1-2 | 1  0  1-5 | 1  0  1-2 |
| **Breathing resistance**  Median  IQR 75-25  Min-Max | 1  0  1-2 | 1  0  1-5 | 1  0  1-2 | 1  0  1-3 | 1  0  1-1 | 1  1  1-3 | 1  1  1-4 | 2  1  1-7 | 1  0  1-2 | 1  1  1-3 | 1  1  1-4 | 2  2  1-6 | 1  0  1-1 | 1  1  1-4 | 1  1  1-4 | 2  1  1-7 | 1  0  1-1 | 1  1  1-3 | 1  1  1-3 | 3  1  1-8 | 1  0  1-1 | 1  1  1-5 | 1  1  1-3 | 2  1  1-9 | 1  0  1-1 | 1  1  1-5 | 1  1  1-3 | 2  2  1-8 | 1  0  1-1 | 1  0  1-2 | 1  0  1-4 | 1  0  1-2 |
| **Itchy**  Median  IQR 75-25  Min-Max | 1  0  1-1 | 1  0  1-6 | 1  0  1-1 | 1  0  1-5 | 1  0  1-1 | 1  0  1-4 | 1  0  1-3 | 1  1  1-5 | 1  0  1-1 | 1  1  1-3 | 1  1  1-3 | 1  1  1-5 | 1  0  1-1 | 1  1  1-4 | 1  1  1-3 | 1  1  1-7 | 1  0  1-1 | 1  1  1-4 | 1  1  1-4 | 1  1  1-6 | 1  0  1-1 | 1  1  1-3 | 1  1  1-3 | 1  2  1-7 | 1  0  1-1 | 1  0  1-4 | 1  1  1-4 | 1  2  1-8 | 1  0  1-1 | 1  0  1-2 | 1  0  1-2 | 1  0  1-2 |
| **Tight**  Median  IQR 75-25  Min-Max | 1  0  1-1 | 1  0  1-3 | 1  0  1-1 | 1  0  1-2 | 1  0  1-1 | 1  0  1-2 | 1  0  1-3 | 2  2  1-6 | 1  0  1-1 | 1  0  1-2 | 1  0  1-3 | 2  2  1-7 | 1  0  1-1 | 1  0  1-2 | 1  0  1-3 | 2  2  1-8 | 1  0  1-1 | 1  0  1-3 | 1  0  1-3 | 2  3  1-10 | 1  0  1-2 | 1  0  1-4 | 1  0  1-3 | 2  3  1-10 | 1  0  1-1 | 1  0  1-4 | 1  0  1-3 | 2  3  1-10 | 1  0  1-1 | 1  0  1-2 | 1  0  1-2 | 1  0  1-2 |
| **Salty**  Median  IQR 75-25  Min-Max | 1  0  1-1 | 1  0  1-1 | 1  0  1-2 | 1  0  1-1 | 1  0  1-1 | 1  0  1-2 | 1  0  1-3 | 1  0  1-4 | 1  0  1-1 | 1  0  1-2 | 1  0  1-3 | 1  0  1-4 | 1  0  1-1 | 1  0  1-4 | 1  0  1-3 | 1  1  1-4 | 1  0  1-1 | 1  0  1-3 | 1  0  1-2 | 1  1  1-5 | 1  0  1-1 | 1  0  1-6 | 1  0  1-3 | 1  1  1-7 | 1  0  1-1 | 1  0  1-5 | 1  0  1-3 | 1  1  1-7 | 1  0  1-1 | 1  0  1-5 | 1  0  1-3 | 1  0  1-2 |
| **Unfit**  Median  IQR 75-25  Min-Max | 1  0  1-1 | 1  0  1-2 | 1  0  1-1 | 1  0  1-2 | 1  0  1-1 | 1  0  1-3 | 1  1  1-8 | 2  2  1-10 | 1  0  1-1 | 1  1  1-10 | 1  1  1-9 | 2  1  1-7 | 1  0  1-1 | 1  1  1-3 | 1  1  1-9 | 2  2  1-10 | 1  0  1-1 | 1  1  1-3 | 1  1  1-9 | 2  2  1-10 | 1  0  1-1 | 1  1  1-3 | 1  1  1-9 | 2  3  1-10 | 1  0  1-1 | 1  1  1-3 | 1  1  1-9 | 2  3  1-10 | 1  0  1-1 | 1  0  1-1 | 1  0  1-8 | 1  0  1-1 |
| **Odour**  Median  IQR 75-25  Min-Max | 1  0  1-2 | 1  0  1-3 | 1  0  1-2 | 1  0  1-2 | 1  0  1-1 | 1  0  1-5 | 1  0  1-7 | 1  1  1-5 | 1  0  1-1 | 1  1  1-6 | 1  1  1-7 | 1  1  1-6 | 1  0  1-1 | 1  1  1-7 | 1  1  1-7 | 1  1  1-6 | 1  0  1-1 | 1  1  1-8 | 1  1  1-5 | 1  2  1-6 | 1  0  1-9 | 1  1  1-7 | 1  1  1-6 | 1  1  1-7 | 1  0  1-1 | 1  1  1-7 | 1  1  1-6 | 2  2  1-8 | 1  0  1-1 | 1  0  1-6 | 1  0  1-5 | 1  0  1-2 |
| **Fatigue**  Median  IQR 75-25  Min-Max | 1  0  1-2 | 1  0  1-6 | 1  0  1-2 | 1  0  1-2 | 1  0  1-2 | 1  1  1-5 | 1  0  1-2 | 1  1  1-6 | 1  0  1-2 | 1  1  1-4 | 1  0  1-3 | 1  1  1-6 | 1  0  1-1 | 1  1  1-6 | 1  1  1-5 | 1  1  1-6 | 1  0  1-2 | 1  1  1-5 | 1  1  1-3 | 2  2  1-7 | 1  0  1-1 | 1  1  1-6 | 1  1  1-3 | 1  2  1-7 | 1  0  1-1 | 1  1  1-10 | 1  1  1-3 | 2  2  1-8 | 1  0  1-1 | 1  0  1-3 | 1  0  1-3 | 1  0  1-4 |
| **Overall discomfort** Median  IQR 75-25  Min-Max | 1  0  1-2 | 1  0  1-4 | 1  0  1-3 | 1  0  1-3 | 1  0  1-3 | 2  1  1-4 | 1  1  1-7 | 3  1  1-8 | 1  0  1-3 | 2  2  1-5 | 2  1  1-6 | 3  2  1-8 | 1  0  1-3 | 2  2  1-6 | 2  2  1-7 | 3  2  1-10 | 1  0  1-3 | 2  2  1-6 | 2  2  1-8 | 3  2  1-10 | 1  0  1-3 | 2  2  1-6 | 2  2  1-8 | 3  3  2-10 | 1  0  1-3 | 2  2  1-6 | 2  1  1-7 | 3  2  1-10 | 1  0  1-2 | 1  0  1-5 | 1  0  1-4 | 1  0  1-3 |
| **Sum***  Median  IQR 75-25  Min-Max | 9  0  9-14 | 9  0  9-32 | 9  0  9-13 | 9  0  9-20 | 9  0  9-10 | 11  3  9-19 | 11  5  9-24 | 15  9  9-44 | 9  0  9-12 | 11  6  9-21 | 11  5  9-25 | 15  8  9-42 | 9  0  9-10 | 12  5  9-24 | 12  7  9-26 | 18  11  10-48 | 9  0  9-10 | 12  5  9-24 | 13  6  9-28 | 19  10  10-51 | 9  0  9-17 | 12  7  9-33 | 12  6  9-29 | 19  12  10-62 | 9  0  9-10 | 12  8  9-37 | 13  7  9-30 | 19  11  9-65 | 9  0  9-10 | 9  0  9-24 | 9  0  9-32 | 9  0  9-17 |

NM: no mask; SM: surgical mask; CM: community mask; FFP2: filtering face piece class 2; CPET: cardiopulmonary exercise test

*The sum includes all scores except for overall discomfort.

Table S2: Results of Symptom Score questionnaire of 40 subjects without mask (NM) and with three different mask types (SM, CM, FFP2) before (pre) and after (post) ergometry (a), CPET (b), and at different timepoints (pre, 30-240 min., post) during workplace examination (c).

| **a) Ergometry** | **Pre** | | | | **Post** | | | |
| --- | --- | --- | --- | --- | --- | --- | --- | --- |
|  | **NM** | **SM** | **CM** | **FFP2** | **NM** | **SM** | **CM** | **FFP2** |
| **Tenseness**  Median  IQR 75-25  Min-Max | 1  0  1-2 | 1  0  1-2 | 1  0  1-2 | 1  0  1-2 | 1  0  1-1 | 1  0  1-2 | 1  0  1-2 | 1  0  1-2 |
| **Anxious**  Median  IQR 75-25  Min-Max | 1  0  1-1 | 1  0  1-1 | 1  0  1-2 | 1  0  1-1 | 1  0  1-3 | 1  0  1-1 | 1  0  1-1 | 1  0  1-1 |
| **Unrest, panic**  Median  IQR 75-25  Min-Max | 1  0  1-1 | 1  0  1-2 | 1  0  1-2 | 1  0  1-2 | 1  0  1-3 | 1  0  1-1 | 1  0  1-1 | 1  0  1-3 |
| **Faster deeper breathing**  Median  IQR 75-25  Min-Max | 1  0  1-2 | 1  0  1-2 | 1  0  1-2 | 1  0  1-2 | 1  0  1-2 | 1  1  1-3 | 1  1  1-4 | 1  1  1-4 |
| **Need of air**  Median  IQR 75-25  Min-Max | 1  0  1-1 | 1  0  1-2 | 1  0  1-1 | 1  0  1-1 | 1  0  1-1 | 1  0  1-2 | 1  0  1-2 | 1  0  1-4 |
| **Chest pressure**  Median  IQR 75-25  Min-Max | 1  0  1-2 | 1  0  1-2 | 1  0  1-2 | 1  0  1-1 | 1  0  1-2 | 1  0  1-2 | 1  0  1-3 | 1  0  1-2 |
| **Suffocating feeling**  Median  IQR 75-25  Min-Max | 1  0  1-1 | 1  0  1-1 | 1  0  1-1 | 1  0  1-1 | 1  0  1-1 | 1  0  1-2 | 1  0  1-1 | 1  0  1-4 |
| **Pounding heart**  Median  IQR 75-25  Min-Max | 1  0  1-2 | 1  0  1-1 | 1  0  1-2 | 1  0  1-2 | 1  0  1-3 | 1  0  1-4 | 1  0  1-3 | 1  1  1-4 |
| **Feeling of heat**  Median  IQR 75-25  Min-Max | 1  0  1-3 | 1  0  1-2 | 1  0  1-2 | 1  0  1-2 | 1  1  1-3 | 2  2  1-4 | 2  2  1-4 | 2  2  1-5 |
| **Head warmth**  Median  IQR 75-25  Min-Max | 1  0  1-1 | 1  0  1-2 | 1  0  1-2 | 1  0  1-2 | 1  1  1-3 | 2  1  1-5 | 1  2  1-4 | 2  2  1-5 |
| **Tingling in arms**  Median  IQR 75-25  Min-Max | 1  0  1-2 | 1  0  1-1 | 1  0  1-1 | 1  0  1-1 | 1  0  1-2 | 1  0  1-5 | 1  0  1-3 | 1  0  1-2 |
| **Tingling in face**  Median  IQR 75-25  Min-Max | 1  0  1-1 | 1  0  1-1 | 1  0  1-1 | 1  0  1-2 | 1  0  1-2 | 1  0  1-2 | 1  0  1-3 | 1  0  1-2 |
| **Knot in throat**  Median  IQR 75-25  Min-Max | 1  0  1-1 | 1  0  1-1 | 1  0  1-1 | 1  0  1-1 | 1  0  1-1 | 1  0  1-1 | 1  0  1-1 | 1  0  1-1 |
| **Headache**  Median  IQR 75-25  Min-Max | 1  0  1-1 | 1  0  1-1 | 1  0  1-2 | 1  0  1-2 | 1  0  1-1 | 1  0  1-1 | 1  0  1-2 | 1  0  1-2 |
| **Dizziness**  Median  IQR 75-25  Min-Max | 1  0  1-2 | 1  0  1-1 | 1  0  1-2 | 1  0  1-1 | 1  0  1-2 | 1  0  1-2 | 1  0  1-3 | 1  0  1-1 |
| **Shivering**  Median  IQR 75-25  Min-Max | 1  0  1-1 | 1  0  1-1 | 1  0  1-1 | 1  0  1-1 | 1  0  1-1 | 1  0  1-1 | 1  0  1-1 | 1  0  1-1 |
| **Joint pain**  Median  IQR 75-25  Min-Max | 1  0  1-1 | 1  0  1-1 | 1  0  1-1 | 1  0  1-1 | 1  0  1-2 | 1  0  1-1 | 1  0  1-1 | 1  0  1-1 |
| **Sleepy feeling**  Median  IQR 75-25  Min-Max | 1  0  1-1 | 1  0  1-3 | 1  0  1-2 | 1  0  1-2 | 1  0  1-3 | 1  0  1-2 | 1  0  1-1 | 1  0  1-1 |
| **Low back pain**  Median  IQR 75-25  Min-Max | 1  0  1-1 | 1  0  1-1 | 1  0  1-1 | 1  0  1-2 | 1  0  1-2 | 1  0  1-1 | 1  0  1-1 | 1  0  1-2 |
| **Burning eyes**  Median  IQR 75-25  Min-Max | 1  0  1-2 | 1  0  1-3 | 1  0  1-4 | 1  0  1-4 | 1  0  1-2 | 1  0  1-3 | 1  0  1-4 | 1  0  1-5 |
| **Sum***  Median  IQR 75-25  Min-Max | 16  0  16-22 | 16  0  16-20 | 16  0  16-22 | 16  1  16-18 | 17  2  16-29 | 18  6  16-26 | 18  4  16-29 | 19  6  16-36 |

| **b) CPET** | **Pre** | | | | **Post** | | | |
| --- | --- | --- | --- | --- | --- | --- | --- | --- |
|  | **NM** | **SM** | **CM** | **FFP2** | **NM** | **SM** | **CM** | **FFP2** |
| **Tenseness**  Median  IQR 75-25  Min-Max | 1  0  1-2 | 1  0  1-2 | 1  0  1-3 | 1  0  1-2 | 1  0  1-3 | 1  0  1-2 | 1  0  1-3 | 1  0  1-2 |
| **Anxious**  Median  IQR 75-25  Min-Max | 1  0  1-2 | 1  0  1-2 | 1  0  1-1 | 1  0  1-2 | 1  0  1-3 | 1  0  1-1 | 1  0  1-2 | 1  0  1-1 |
| **Unrest, panic**  Median  IQR 75-25  Min-Max | 1  0  1-2 | 1  0  1-2 | 1  0  1-2 | 1  0  1-2 | 1  0  1-3 | 1  0  1-1 | 1  0  1-3 | 1  0  1-2 |
| **Faster deeper breathing**  Median  IQR 75-25  Min-Max | 1  0  1-2 | 1  0  1-2 | 1  0  1-2 | 1  0  1-3 | 1  0  1-3 | 1  1  1-3 | 1  1  1-3 | 2  1  1-4 |
| **Need of air**  Median  IQR 75-25  Min-Max | 1  0  1-2 | 1  0  1-2 | 1  0  1-2 | 1  0  1-4 | 1  0  1-2 | 1  1  1-3 | 1  1  1-3 | 1  1  1-5 |
| **Chest pressure**  Median  IQR 75-25  Min-Max | 1  0  1-1 | 1  0  1-2 | 1  0  1-1 | 1  0  1-2 | 1  0  1-2 | 1  0  1-2 | 1  0  1-1 | 1  0  1-2 |
| **Suffocating feeling**  Median  IQR 75-25  Min-Max | 1  0  1-1 | 1  0  1-2 | 1  0  1-2 | 1  0  1-2 | 1  0  1-2 | 1  0  1-2 | 1  0  1-2 | 1  0  1-5 |
| **Pounding heart**  Median  IQR 75-25  Min-Max | 1  0  1-2 | 1  0  1-1 | 1  0  1-2 | 1  0  1-2 | 1  0  1-4 | 1  1  1-4 | 1  1  1-3 | 1  1  1-5 |
| **Feeling of heat**  Median  IQR 75-25  Min-Max | 1  0  1-2 | 1  0  1-2 | 1  0  1-3 | 1  0  1-3 | 2  2  1-4 | 2  2  1-4 | 2  2  1-5 | 2  1  1-5 |
| **Head warmth**  Median  IQR 75-25  Min-Max | 1  0  1-2 | 1  0  1-2 | 1  0  1-3 | 1  0  1-3 | 2  1  1-4 | 2  2  1-4 | 2  2  1-4 | 2  1  1-5 |
| **Tingling in arms**  Median  IQR 75-25  Min-Max | 1  0  1-1 | 1  0  1-1 | 1  0  1-1 | 1  0  1-1 | 1  0  1-2 | 1  0  1-3 | 1  0  1-2 | 1  0  1-2 |
| **Tingling in face**  Median  IQR 75-25  Min-Max | 1  0  1-1 | 1  0  1-2 | 1  0  1-2 | 1  0  1-2 | 1  0  1-2 | 1  0  1-2 | 1  0  1-3 | 1  0  1-3 |
| **Knot in throat**  Median  IQR 75-25  Min-Max | 1  0  1-1 | 1  0  1-2 | 1  0  1-1 | 1  0  1-1 | 1  0  1-2 | 1  0  1-3 | 1  0  1-1 | 1  0  1-2 |
| **Headache**  Median  IQR 75-25  Min-Max | 1  0  1-2 | 1  0  1-2 | 1  0  1-3 | 1  0  1-2 | 1  0  1-3 | 1  0  1-3 | 1  0  1-2 | 1  0  1-3 |
| **Dizziness**  Median  IQR 75-25  Min-Max | 1  0  1-1 | 1  0  1-1 | 1  0  1-2 | 1  0  1-1 | 1  0  1-3 | 1  0  1-2 | 1  0  1-2 | 1  0  1-2 |
| **Shivering**  Median  IQR 75-25  Min-Max | 1  0  1-1 | 1  0  1-1 | 1  0  1-1 | 1  0  1-1 | 1  0  1-1 | 1  0  1-1 | 1  0  1-1 | 1  0  1-1 |
| **Joint pain**  Median  IQR 75-25  Min-Max | 1  0  1-1 | 1  0  1-1 | 1  0  1-1 | 1  0  1-1 | 1  0  1-1 | 1  0  1-1 | 1  0  1-2 | 1  0  1-1 |
| **Sleepy feeling**  Median  IQR 75-25  Min-Max | 1  0  1-2 | 1  0  1-1 | 1  0  1-2 | 1  0  1-2 | 1  0  1-2 | 1  0  1-2 | 1  0  1-2 | 1  0  1-2 |
| **Low back pain**  Median  IQR 75-25  Min-Max | 1  0  1-1 | 1  0  1-2 | 1  0  1-1 | 1  0  1-1 | 1  0  1-2 | 1  0  1-1 | 1  0  1-2 | 1  0  1-1 |
| **Burning eyes**  Median  IQR 75-25  Min-Max | 1  0  1-1 | 1  0  1-1 | 1  0  1-1 | 1  0  1-1 | 1  0  1-1 | 1  0  1-2 | 1  0  1-1 | 1  0  1-1 |
| **Sum***  Median  IQR 75-25  Min-Max | 16  1  16-19 | 16  1  16-22 | 16  2  16-21 | 16  2  16-28 | 19  4  16-30 | 19  7  16-34 | 18  5  16-31 | 20  6  16-36 |

| **c)**  **Workplace**  **examination** | Pre | | | | 30 | | | | 60 | | | | 90 | | | | 150 | | | | 210 | | | | 240 | | | | Post | | | |
| --- | --- | --- | --- | --- | --- | --- | --- | --- | --- | --- | --- | --- | --- | --- | --- | --- | --- | --- | --- | --- | --- | --- | --- | --- | --- | --- | --- | --- | --- | --- | --- | --- |
|  | **NM** | **SM** | **CM** | **FFP** | **NM** | **SM** | **CM** | **FFP** | **NM** | **SM** | **CM** | **FFP** | **NM** | **SM** | **CM** | **FFP** | **NM** | **SM** | **CM** | **FFP** | **NM** | **SM** | **CM** | **FFP** | **NM** | **SM** | **CM** | **FFP** | **NM** | **SM** | **CM** | **FFP** |
| **Tenseness**  Median  IQR 75-25  Min-Max | 1  0  1-3 | 1  0  1-2 | 1  0  1-3 | 1  0  1-2 | 1  0  1-3 | 1  1  1-2 | 1  0  1-3 | 1  1  1-3 | 1  0  1-2 | 1  0  1-3 | 1  0  1-3 | 1  0  1-3 | 1  0  1-3 | 1  0  1-3 | 1  0  1-3 | 1  1  1-2 | 1  0  1-3 | 1  1  1-2 | 1  0  1-2 | 1  1  1-2 | 1  0  1-3 | 1  1  1-2 | 1  0  1-2 | 1  1  1-4 | 1  0  1-3 | 1  1  1-2 | 1  0  1-3 | 1  1  1-3 | 1  0  1-2 | 1  0  1-2 | 1  0  1-2 | 1  0  1-3 |
| **Anxious**  Median  IQR 75-25  Min-Max | 1  0  1-1 | 1  0  1-2 | 1  0  1-1 | 1  0  1-2 | 1  0  1-1 | 1  0  1-1 | 1  0  1-1 | 1  0  1-2 | 1  0  1-1 | 1  0  1-1 | 1  0  1-1 | 1  0  1-2 | 1  0  1-1 | 1  0  1-2 | 1  0  1-1 | 1  0  1-2 | 1  0  1-1 | 1  0  1-1 | 1  0  1-1 | 1  0  1-2 | 1  0  1-1 | 1  0  1-2 | 1  0  1-1 | 1  0  1-2 | 1  0  1-1 | 1  0  1-2 | 1  0  1-1 | 1  0  1-2 | 1  0  1-1 | 1  0  1-1 | 1  0  1-1 | 1  0  1-1 |
| **Unrest, panic**  Median  IQR 75-25  Min-Max | 1  0  1-1 | 1  0  1-2 | 1  0  1-2 | 1  0  1-2 | 1  0  1-1 | 1  0  1-2 | 1  0  1-2 | 1  0  1-2 | 1  0  1-1 | 1  0  1-2 | 1  0  1-2 | 1  0  1-2 | 1  0  1-2 | 1  0  1-2 | 1  0  1-2 | 1  0  1-2 | 1  0  1-1 | 1  0  1-2 | 1  0  1-2 | 1  0  1-3 | 1  0  1-2 | 1  0  1-2 | 1  0  1-2 | 1  0  1-3 | 1  0  1-2 | 1  0  1-2 | 1  0  1-2 | 1  0  1-4 | 1  0  1-1 | 1  0  1-1 | 1  0  1-1 | 1  0  1-3 |
| **Faster deeper breathing**  Median  IQR 75-25  Min-Max | 1  0  1-2 | 1  0  1-3 | 1  0  1-2 | 1  0  1-2 | 1  0  1-1 | 1  0  1-2 | 1  0  1-2 | 1  1  1-3 | 1  0  1-2 | 1  0  1-2 | 1  0  1-2 | 1  1  1-3 | 1  0  1-2 | 1  0  1-3 | 1  0  1-2 | 1  1  1-3 | 1  0  1-2 | 1  0  1-3 | 1  0  1-2 | 1  1  1-3 | 1  0  1-2 | 1  0  1-3 | 1  0  1-2 | 1  1  1-4 | 1  0  1-2 | 1  0  1-3 | 1  0  1-3 | 1  1  1-4 | 1  0  1-2 | 1  0  1-2 | 1  0  1-3 | 1  0  1-2 |
| **Need of air**  Median  IQR 75-25  Min-Max | 1  0  1-1 | 1  0  1-2 | 1  0  1-1 | 1  0  1-1 | 1  0  1-1 | 1  0  1-1 | 1  0  1-2 | 1  0  1-3 | 1  0  1-1 | 1  0  1-2 | 1  0  1-2 | 1  0  1-3 | 1  0  1-1 | 1  0  1-2 | 1  0  1-1 | 1  1  1-2 | 1  0  1-1 | 1  0  1-2 | 1  0  1-1 | 1  1  1-3 | 1  0  1-1 | 1  0  1-3 | 1  0  1-2 | 1  1  1-3 | 1  0  1-1 | 1  0  1-3 | 1  0  1-2 | 1  1  1-3 | 1  0  1-1 | 1  0  1-2 | 1  0  1-1 | 1  0  1-2 |
| **Chest pressure**  Median  IQR 75-25  Min-Max | 1  0  1-1 | 1  0  1-2 | 1  0  1-2 | 1  0  1-1 | 1  0  1-2 | 1  0  1-2 | 1  0  1-2 | 1  0  1-2 | 1  0  1-1 | 1  0  1-2 | 1  0  1-2 | 1  0  1-2 | 1  0  1-2 | 1  0  1-2 | 1  0  1-2 | 1  0  1-2 | 1  0  1-1 | 1  0  1-2 | 1  0  1-2 | 1  0  1-2 | 1  0  1-2 | 1  0  1-3 | 1  0  1-3 | 1  0  1-3 | 1  0  1-2 | 1  0  1-3 | 1  0  1-3 | 1  0  1-3 | 1  0  1-1 | 1  0  1-2 | 1  0  1-3 | 1  0  1-2 |
| **Suffocating feeling**  Median  IQR 75-25  Min-Max | 1  0  1-1 | 1  0  1-1 | 1  0  1-1 | 1  0  1-1 | 1  0  1-1 | 1  0  1-1 | 1  0  1-1 | 1  0  1-2 | 1  0  1-1 | 1  0  1-1 | 1  0  1-1 | 1  0  1-2 | 1  0  1-2 | 1  0  1-2 | 1  0  1-1 | 1  0  1-2 | 1  0  1-1 | 1  0  1-2 | 1  0  1-1 | 1  0  1-3 | 1  0  1-1 | 1  0  1-2 | 1  0  1-1 | 1  0  1-3 | 1  0  1-1 | 1  0  1-2 | 1  0  1-1 | 1  0  1-4 | 1  0  1-1 | 1  0  1-1 | 1  0  1-1 | 1  0  1-1 |
| **Pounding heart**  Median  IQR 75-25  Min-Max | 1  0  1-1 | 1  0  1-2 | 1  0  1-2 | 1  0  1-1 | 1  0  1-1 | 1  0  1-2 | 1  0  1-2 | 1  0  1-2 | 1  0  1-1 | 1  0  1-2 | 1  0  1-2 | 1  0  1-2 | 1  0  1-1 | 1  0  1-2 | 1  0  1-2 | 1  0  1-2 | 1  0  1-1 | 1  0  1-2 | 1  0  1-2 | 1  0  1-3 | 1  0  1-2 | 1  0  1-2 | 1  0  1-2 | 1  0  1-4 | 1  0  1-2 | 1  0  1-2 | 1  0  1-2 | 1  0  1-4 | 1  0  1-1 | 1  0  1-2 | 1  0  1-2 | 1  0  1-2 |
| **Feeling of heat**  Median  IQR 75-25  Min-Max | 1  0  1-2 | 1  0  1-2 | 1  0  1-2 | 1  0  1-3 | 1  0  1-2 | 1  0  1-2 | 1  0  1-2 | 1  0  1-4 | 1  0  1-2 | 1  0  1-4 | 1  0  1-3 | 1  1  1-4 | 1  0  1-2 | 1  1  1-3 | 1  1  1-3 | 1  1  1-4 | 1  0  1-2 | 1  0  1-3 | 1  0  1-4 | 1  1  1-4 | 1  0  1-2 | 1  0  1-3 | 1  1  1-3 | 1  1  1-5 | 1  0  1-2 | 1  0  1-3 | 1  0  1-3 | 1  1  1-5 | 1  0  1-2 | 1  0  1-3 | 1  0  1-3 | 1  0  1-2 |
| **Head warmth**  Median  IQR 75-25  Min-Max | 1  0  1-2 | 1  0  1-2 | 1  0  1-2 | 1  0  1-2 | 1  0  1-1 | 1  0  1-2 | 1  0  1-2 | 1  0  1-2 | 1  0  1-1 | 1  0  1-3 | 1  0  1-3 | 1  0  1-2 | 1  0  1-2 | 1  0  1-3 | 1  0  1-3 | 1  0  1-2 | 1  0  1-2 | 1  0  1-3 | 1  0  1-4 | 1  1  1-3 | 1  0  1-2 | 1  0  1-3 | 1  0  1-3 | 1  1  1-4 | 1  0  1-2 | 1  0  1-3 | 1  0  1-3 | 1  1  1-4 | 1  0  1-2 | 1  0  1-3 | 1  0  1-1 | 1  0  1-3 |
| **Tingling arms**  Median  IQR 75-25  Min-Max | 1  0  1-1 | 1  0  1-1 | 1  0  1-1 | 1  0  1-2 | 1  0  1-1 | 1  0  1-1 | 1  0  1-1 | 1  0  1-2 | 1  0  1-1 | 1  0  1-1 | 1  0  1-1 | 1  0  1-2 | 1  0  1-1 | 1  0  1-1 | 1  0  1-1 | 1  0  1-2 | 1  0  1-2 | 1  0  1-2 | 1  0  1-1 | 1  0  1-2 | 1  0  1-1 | 1  0  1-1 | 1  0  1-1 | 1  0  1-2 | 1  0  1-1 | 1  0  1-1 | 1  0  1-2 | 1  0  1-3 | 1  0  1-1 | 1  0  1-1 | 1  0  1-1 | 1  0  1-2 |
| **Tingling face**  Median  IQR 75-25  Min-Max | 1  0  1-1 | 1  0  1-2 | 1  0  1-1 | 1  0  1-1 | 1  0  1-2 | 1  0  1-1 | 1  0  1-1 | 1  0  1-2 | 1  0  1-2 | 1  0  1-2 | 1  0  1-3 | 1  0  1-2 | 1  0  1-2 | 1  0  1-2 | 1  0  1-2 | 1  0  1-2 | 1  0  1-2 | 1  0  1-2 | 1  0  1-3 | 1  0  1-2 | 1  0  1-2 | 1  0  1-2 | 1  0  1-2 | 1  0  1-3 | 1  0  1-2 | 1  0  1-3 | 1  0  1-2 | 1  0  1-2 | 1  0  1-2 | 1  0  1-2 | 1  0  1-1 | 1  0  1-2 |
| **Knot in throat**  Median  IQR 75-25  Min-Max | 1  0  1-1 | 1  0  1-1 | 1  0  1-2 | 1  0  1-1 | 1  0  1-1 | 1  0  1-1 | 1  0  1-1 | 1  0  1-2 | 1  0  1-1 | 1  0  1-1 | 1  0  1-2 | 1  0  1-2 | 1  0  1-1 | 1  0  1-2 | 1  0  1-2 | 1  0  1-2 | 1  0  1-1 | 1  0  1-2 | 1  0  1-2 | 1  0  1-2 | 1  0  1-1 | 1  0  1-2 | 1  0  1-1 | 1  0  1-2 | 1  0  1-1 | 1  0  1-2 | 1  0  1-1 | 1  0  1-2 | 1  0  1-1 | 1  0  1-2 | 1  0  1-1 | 1  0  1-2 |
| **Headache**  Median  IQR 75-25  Min-Max | 1  0  1-2 | 1  0  1-2 | 1  0  1-1 | 1  0  1-2 | 1  0  1-2 | 1  0  1-2 | 1  0  1-2 | 1  0  1-3 | 1  0  1-2 | 1  0  1-3 | 1  0  1-2 | 1  0  1-4 | 1  0  1-2 | 1  0  1-4 | 1  0  1-3 | 1  0  1-4 | 1  0  1-1 | 1  0  1-3 | 1  0  1-3 | 1  0  1-4 | 1  0  1-1 | 1  1  1-4 | 1  0  1-3 | 1  0  1-4 | 1  0  1-1 | 1  0  1-4 | 1  0  1-3 | 1  0  1-4 | 1  0  1-1 | 1  0  1-3 | 1  0  1-2 | 1  0  1-2 |
| **Dizziness**  Median  IQR 75-25  Min-Max | 1  0  1-1 | 1  0  1-2 | 1  0  1-2 | 1  0  1-2 | 1  0  1-1 | 1  0  1-2 | 1  0  1-1 | 1  0  1-2 | 1  0  1-1 | 1  0  1-2 | 1  0  1-1 | 1  0  1-2 | 1  0  1-1 | 1  0  1-2 | 1  0  1-1 | 1  0  1-2 | 1  0  1-1 | 1  0  1-2 | 1  0  1-1 | 1  0  1-3 | 1  0  1-1 | 1  0  1-2 | 1  0  1-1 | 1  0  1-3 | 1  0  1-1 | 1  0  1-3 | 1  0  1-2 | 1  0  1-4 | 1  0  1-1 | 1  0  1-3 | 1  0  1-2 | 1  0  1-2 |
| **Shivering**  Median  IQR 75-25  Min-Max | 1  0  1-1 | 1  0  1-1 | 1  0  1-1 | 1  0  1-1 | 1  0  1-1 | 1  0  1-1 | 1  0  1-1 | 1  0  1-1 | 1  0  1-1 | 1  0  1-1 | 1  0  1-1 | 1  0  1-2 | 1  0  1-1 | 1  0  1-1 | 1  0  1-1 | 1  0  1-1 | 1  0  1-1 | 1  0  1-1 | 1  0  1-1 | 1  0  1-1 | 1  0  1-1 | 1  0  1-1 | 1  0  1-3 | 1  0  1-1 | 1  0  1-1 | 1  0  1-1 | 1  0  1-1 | 1  0  1-1 | 1  0  1-1 | 1  0  1-1 | 1  0  1-1 | 1  0  1-1 |
| **Joint pain**  Median  IQR 75-25  Min-Max | 1  0  1-3 | 1  0  1-1 | 1  0  1-3 | 1  0  1-1 | 1  0  1-3 | 1  0  1-1 | 1  0  1-3 | 1  0  1-3 | 1  0  1-3 | 1  0  1-1 | 1  0  1-3 | 1  0  1-2 | 1  0  1-3 | 1  0  1-3 | 1  0  1-3 | 1  0  1-3 | 1  0  1-3 | 1  0  1-2 | 1  0  1-3 | 1  0  1-3 | 1  0  1-3 | 1  0  1-2 | 1  0  1-1 | 1  0  1-1 | 1  0  1-3 | 1  0  1-3 | 1  0  1-3 | 1  0  1-1 | 1  0  1-3 | 1  0  1-2 | 1  0  1-3 | 1  0  1-1 |
| **Sleepy feeling**  Median  IQR 75-25  Min-Max | 1  0  1-3 | 1  0  1-2 | 1  0  1-1 | 1  0  1-2 | 1  0  1-3 | 1  0  1-3 | 1  0  1-2 | 1  0  1-2 | 1  0  1-2 | 1  0  1-2 | 1  0  1-3 | 1  0  1-3 | 1  0  1-2 | 1  0  1-3 | 1  0  1-3 | 1  0  1-3 | 1  0  1-1 | 1  0  1-3 | 1  0  1-2 | 1  1  1-4 | 1  0  1-2 | 1  0  1-4 | 1  0  1-2 | 1  1  1-3 | 1  0  1-2 | 1  0  1-3 | 1  0  1-3 | 1  1  1-4 | 1  0  1-1 | 1  0  1-3 | 1  1  1-3 | 1  0  1-2 |
| **Low back pain**  Median  IQR 75-25  Min-Max | 1  0  1-1 | 1  0  1-2 | 1  0  1-1 | 1  0  1-1 | 1  0  1-1 | 1  0  1-2 | 1  0  1-1 | 1  0  1-2 | 1  0  1-1 | 1  0  1-2 | 1  0  1-1 | 1  0  1-5 | 1  0  1-1 | 1  0  1-2 | 1  0  1-2 | 1  0  1-1 | 1  0  1-1 | 1  0  1-2 | 1  0  1-2 | 1  0  1-2 | 1  0  1-2 | 1  0  1-2 | 1  0  1-2 | 1  0  1-2 | 1  0  1-1 | 1  0  1-2 | 1  0  1-2 | 1  0  1-3 | 1  0  1-1 | 1  0  1-2 | 1  0  1-2 | 1  0  1-2 |
| **Burning eyes**  Median  IQR 75-25  Min-Max | 1  0  1-1 | 1  0  1-2 | 1  0  1-1 | 1  0  1-1 | 1  0  1-1 | 1  0  1-3 | 1  0  1-2 | 1  0  1-2 | 1  0  1-1 | 1  0  1-3 | 1  0  1-2 | 1  0  1-2 | 1  0  1-1 | 1  0  1-3 | 1  0  1-2 | 1  0  1-2 | 1  0  1-2 | 1  0  1-3 | 1  0  1-2 | 1  0  1-2 | 1  0  1-3 | 1  0  1-3 | 1  0  1-2 | 1  0  1-2 | 1  0  1-1 | 1  0  1-3 | 1  0  1-2 | 1  0  1-3 | 1  0  1-1 | 1  0  1-3 | 1  0  1-1 | 1  0  1-2 |
| **Sum***  Median  IQR 75-25  Min-Max | 16  0  16-19 | 16  0  16-26 | 16  0  16-22 | 16  0  16-24 | 16  0  16-18 | 16  1  16-24 | 16  1  16-23 | 17  2  16-31 | 16  0  16-18 | 16  2  16-25 | 16  1  16-25 | 17  3  16-32 | 16  0  16-19 | 16  2  16-27 | 16  2  16-24 | 18  3  16-31 | 16  0  16-19 | 16  2  16-29 | 16  2  16-28 | 19  4  16-35 | 16  0  16-20 | 16  3  16-33 | 16  2  16-24 | 19  4  16-43 | 16  0  16-20 | 16  3  16-31 | 16  2  16-28 | 18  5  16-44 | 16  0  16-18 | 16  0  16-27 | 16  1  16-22 | 16  0  16-26 |

NM: no mask; SM: surgical mask; CM: community mask; FFP2: filtering face piece class 2; CPET: cardiopulmonary exercise test

*The sum includes all scores except for joint pain, sleepy feeling, low back pain, and burning eyes.
