# Supplementary figures and images for: Influence of face masks on the subjective impairment at different physical workloads
Source: Sci Rep. 2023 May 19;13:8133. doi: 10.1038/s41598-023-34319-0 (PMC10196290; doi:10.1038/s41598-023-34319-0)

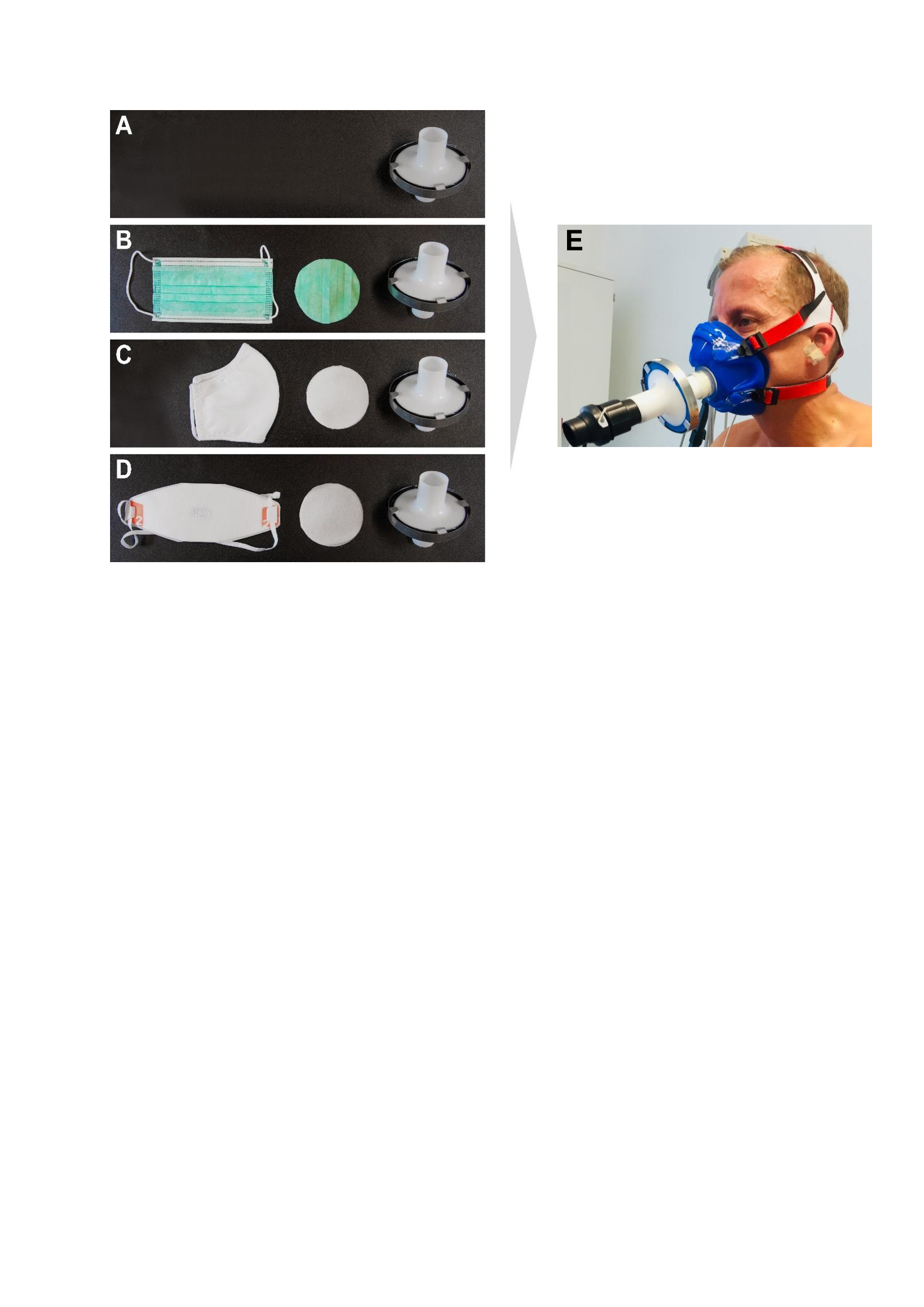

Supplement: Supplementary file 2 — Supplementary Figure S1. [file 41598_2023_34319_MOESM2_ESM.jpg]
