# Supplementary material for: Influence of face masks on the subjective impairment at different physical workloads
Source: Sci Rep. 2023 May 19;13:8133. doi: 10.1038/s41598-023-34319-0 (PMC10196290; doi:10.1038/s41598-023-34319-0)
Supplement: Supplementary file 3 — Supplementary Legends. [file 41598_2023_34319_MOESM3_ESM.docx]

**Figure 1: Mask adapter**. A round material sample (Ø 8 cm) of the tested mask ((B) surgical mask, (C) community mask, (D) FFP2 mask) was placed in an opened, empty bacterial filter, which was then airtight closed and used for cardiopulmonary exercise test (E). For no mask situation (A) an empty filter housing was used.
